# An audiovisual assistance system for beam-stopper positioning in intraoperative electron radiotherapy: experimental evaluation under manual and teleoperated operation

**DOI:** 10.3389/frobt.2026.1955069

**Published:** 2026-10-30

**Authors:** Laura Garrido-Rey, Silvia Casans, José Manuel Deltoro, Irene del Canto, Enrique Sanchis, Vicent Girbés-Juan

**Affiliations:** 1 Instituto de Física Corpuscular, Centro Superior de Investigaciones Científicas y Universitat de València, Paterna, Spain; 2 Departamento de Ingeniería Electrónica, Universitat de València, Burjassot, Spain

**Keywords:** audiovisual feedback, cognitive workload, human–robot interaction, IOERT, multimodal assistance, NASA-TLX, robotic guidance, teleoperation

## Abstract

**Introduction:**

Robotic positioning tasks involving indirectly observable targets present significant spatial reasoning challenges in human–robot interaction (HRI). A clinically relevant example is beam stopper positioning during intraoperative electron radiotherapy (IOERT), which requires aligning a mobile shielding device with an unseen radiation beam axis.

**Methods:**

We propose an audiovisual assistance system that estimates the real-time intersection between the radiation beam and the floor plane, providing multimodal spatial guidance during both manual and teleoperated control. A within-subject experimental study was conducted to evaluate positioning accuracy, execution time, user experience, and workload across assisted and unassisted conditions.

**Results:**

Audiovisual guidance reduced the mean positioning error by approximately 80% (from ∼ 0.30 m to less than 0.07 m) across both control modes without increasing completion time. Furthermore, the system mitigated performance degradation caused by unfavorable workspace geometries, minimized skill-based differences between operators, and significantly lowered perceived mental workload.

**Discussion:**

These findings demonstrate that real-time multimodal feedback is a robust, effective strategy for improving spatial accuracy, consistency, and usability in HRI tasks with obscured target geometries.

## 1 Introduction

Human–robot interaction (HRI) investigates the design, implementation and evaluation of robotic systems that enable effective, safe and intuitive collaboration between humans and robots in shared tasks and environments (Goodrich and Schultz, 2008). As robotic technologies continue to expand beyond structured industrial settings toward healthcare, service robotics and other human-centered applications, the operator has become an integral component of the control loop rather than a mere supervisor. Consequently, the design of interaction strategies that improve usability, situational awareness and task performance has become a central objective of contemporary HRI research.

A major challenge in HRI arises in applications requiring precise positioning relative to targets that cannot be directly observed. In these situations, operators must infer the spatial relationship between the robot and an invisible or partially occluded target by integrating indirect geometric information with their own perception of the environment (Sheridan, 1992). Such tasks impose considerable perceptual and cognitive demands, particularly when they involve three-dimensional spatial reasoning, restricted workspaces or limited visual feedback (Abbink et al., 2018; Pan et al., 2024). These conditions are frequently encountered in robotic manipulation, medical robotics, industrial inspection and teleoperated systems, where accurate positioning depends not only on robot control but also on the operator’s ability to interpret incomplete environmental information (Wang et al., 2024; Su et al., 2023).

To support operators in these demanding scenarios, increasing attention has been devoted to shared-control and human-centered assistance strategies. Rather than replacing human decision making, these approaches aim to combine the complementary capabilities of humans and robots by allowing the robot to provide guidance or task-specific assistance while preserving human authority over task execution (Abbink et al., 2018; Pan et al., 2024). Within this framework, assistance systems are intended to reduce operator workload, improve positioning accuracy and facilitate task execution without compromising user control (Losey et al., 2018).

Among the different assistance strategies proposed in HRI, multimodal interaction has emerged as an effective mechanism for improving human performance in complex robotic tasks. By combining complementary communication channels, multimodal HAV (haptic-audio-visual) interfaces can present task-relevant information in ways that better match human perceptual capabilities, thereby improving spatial understanding, operator awareness and decision making (Enayati et al., 2016; Su et al., 2023). In particular, visual feedback provides intuitive spatial information, whereas auditory cues can continuously convey directional or positional guidance without increasing visual demand (Girbes et al., 2016). Their combination has been shown to improve task efficiency and reduce cognitive effort in a wide range of robot-assisted applications (Girbes-Juan et al., 2021; Girbés-Juan et al., 2022).

These advantages become particularly relevant in teleoperation, where the physical separation between the operator and the robot often reduces direct perception of the environment. Although teleoperation enables robotic operation in hazardous, inaccessible or constrained workspaces (Sheridan, 1992; Wang et al., 2024), indirect interaction may degrade situational awareness and make spatial alignment tasks more challenging than direct manipulation. Consequently, considerable research has focused on developing interaction techniques capable of compensating for these perceptual limitations through enhanced feedback modalities and operator assistance (Su et al., 2023). Recent studies have demonstrated that multimodal interfaces improve teleoperation performance and positioning accuracy (Girbés-Juan et al., 2022; Mazeas and Namoano, 2025), while augmented visualization and audiovisual feedback have also been shown to enhance user experience and reduce cognitive workload (Tecco et al., 2025).

Although these advances have been successfully applied to robotic manipulation, navigation and industrial inspection, comparatively fewer studies have addressed positioning problems in which the target itself cannot be directly observed and must instead be inferred from geometric relationships within the workspace. This class of problems remains particularly challenging because it requires operators to continuously estimate the location of an invisible target while simultaneously controlling robot motion, increasing perceptual and cognitive demands (Abbink et al., 2018; Pan et al., 2024). Such situations frequently arise in medical robotics (Dupont et al., 2021), where safety requirements, constrained workspaces and limited accessibility further increase task complexity (Hensley, 2017; Wang et al., 2024).

Intraoperative electron radiotherapy (IOERT) constitutes a representative example of this type of interaction problem. IOERT enables the delivery of a high radiation dose directly to the tumor bed immediately after surgical resection while minimizing irradiation of surrounding healthy tissues and has become an established treatment option for several oncological indications, including breast cancer (Roeder et al., 2020), gynecological malignancies (Cornejo et al., 2026), soft tissue sarcomas (Grimm et al., 2024), and pancreatic cancer (Erdemoglu et al., 2025). The introduction of mobile linear accelerators has further expanded the applicability of IOERT by enabling treatment delivery in conventional operating rooms without requiring dedicated radiation facilities.

Unlike conventional external beam radiotherapy, IOERT is performed in a dynamic and spatially constrained clinical environment in which surgeons, radiation oncologists, medical physicists and multiple medical devices simultaneously share the workspace (Hensley, 2017). Beyond the dosimetric performance of the accelerator itself, treatment quality and safety depend on the accurate deployment of auxiliary devices that ensure adequate radiation protection during irradiation.

Among the mobile accelerators currently employed for IOERT, the LIAC HWL system incorporates a mobile shielding device, commonly referred to as a beam stopper, which is positioned beneath the operating table to attenuate the primary electron beam and reduce radiation transmitted to lower floors of the hospital building (S.I.T. Sordina IORT Technologies S.p.A, 2026; Winkler et al., 2020). Although the beam stopper provides a substantial geometric safety margin due to its dimensions and shielding characteristics, its effectiveness ultimately depends on its correct alignment with the beam axis. Consequently, beam stopper positioning constitutes a critical step in treatment preparation.

From an HRI perspective, this procedure can be interpreted as a spatial alignment task in which the operator must position the robotic platform with respect to a target that cannot be directly observed. Because the point where the radiation beam intersects the floor plane is invisible, operators must infer its location from the accelerator pose while simultaneously coping with restricted accessibility beneath the operating table and limited visibility caused by surrounding equipment and clinical personnel. The task therefore combines the perceptual challenges associated with indirect target localization with the operational constraints characteristic of clinical environments.

Despite the extensive literature devoted to IOERT dosimetry, radiation protection and accelerator characterization (Hensley, 2017; Winkler et al., 2020; Soriani et al., 2010), comparatively little attention has been paid to the interaction strategies that support operators during beam stopper positioning. Likewise, although multimodal feedback and teleoperation have demonstrated clear benefits in robotic manipulation and navigation tasks (Abbink et al., 2018; Girbes-Juan et al., 2021; Pan et al., 2024), their application to clinically inspired spatial alignment problems involving indirectly observable targets remains largely unexplored. To the best of the authors’ knowledge, no previous work has investigated the use of continuous audiovisual feedback to assist beam stopper positioning during IOERT, nor evaluated its effectiveness under both manual and teleoperated operation while considering objective performance and operator cognitive workload.

To address this gap, this work presents an audiovisual assistance system designed to support beam stopper positioning during IOERT procedures. The proposed approach combines geometric modeling of the radiation beam with robotic localization techniques to estimate in real time the intersection between the beam axis and the floor plane. This estimated target location is then transformed into continuous visual and auditory cues that guide the operator during the positioning process while preserving full human control over robot motion.

The proposed system was evaluated through a within-subject experimental study comparing manual and teleoperated beam stopper positioning under assisted and non-assisted conditions. Performance was assessed using objective metrics, including positioning accuracy and task completion time, together with subjective measures of user experience and cognitive workload obtained through some questionnaires and the NASA Task Load Index (NASA-TLX) (Hart and Staveland, 1988). By analyzing both interaction modalities under identical experimental conditions, this study provides insight into the contribution of audiovisual guidance to human performance during clinically relevant robotic positioning tasks.

The main contributions of this work are threefold: (i) the formulation of beam stopper positioning during IOERT as a robotic spatial alignment problem involving an indirectly observable target, providing an HRI perspective for this clinically relevant task; (ii) the development of an audiovisual assistance system that provides continuous visual and auditory guidance while preserving full operator control during both manual and teleoperated positioning; and (iii) a comprehensive experimental validation demonstrating that audiovisual guidance substantially improves positioning accuracy and reduces operator workload in both manual and teleoperated beam stopper positioning without increasing task completion time.

The remainder of this paper is organized as follows. Section 2 introduces the robotic system and formulates the beam stopper positioning problem. Section 3 presents the proposed audiovisual assistance system together with the experimental methodology. Section 4 reports the experimental results. Section 5 discusses the findings and their implications for HRI in medical robotics. Finally, Section 6 concludes the paper and outlines future research directions.

## 2 System overview and problem formulation

The proposed assistance system addresses beam stopper positioning by estimating the location of the radiation beam on the operating-room floor and guiding the alignment of the shielding device. To enable controlled and repeatable experimentation, the clinical positioning procedure is reproduced using two robotic platforms representing the accelerator and the beam stopper.

Figure 1 shows the robotic models employed throughout this work. The LIAC HWL accelerator is represented by a mobile manipulator M , whose end-effector defines the beam axis, whereas the beam stopper is represented by a differential-drive mobile robot R . The target position is defined by the intersection between the beam axis and the floor plane and is computed from the kinematic model of the manipulator. The objective of the mobile robot is to accurately reach this estimated position.

**FIGURE 1:**
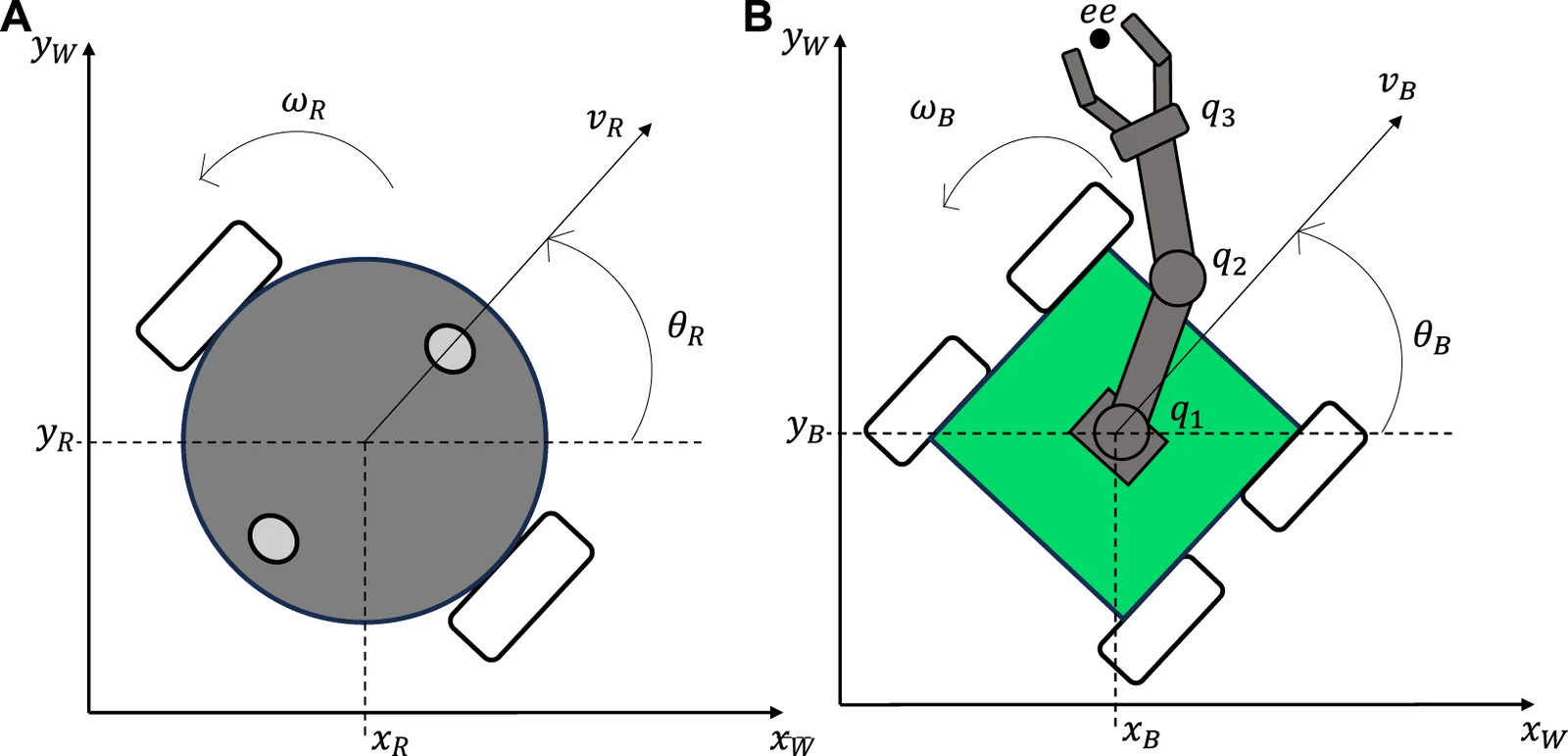
Robotic systems for the beam stopper positioning: **(A)** Non-holonomic mobile robot R as beam stopper, **(B)** LIAC HWL accelerator M modeled as a robotic manipulator mounted on a mobile base.

Based on these robotic models, beam stopper positioning can be formulated as a spatial alignment problem. The following subsection presents the mathematical formulation adopted throughout this work.

### 2.1 Problem statement

Let R be a non-holonomic mobile robot moving on a planar environment, its state space is defined as $q_{R}\left(t\right)={\left[x_{R}\left(t\right)\;\;y_{R}\left(t\right)\;\;\theta_{R}\left(t\right)\right]}^{T}\in R^{2}\times S^{1}$ containing the Cartesian position of the mobile platform, $x_{R}\left(t\right)$ and $y_{R}\left(t\right)$ , and its orientation $\theta_{R}\left(t\right)$ with respect to the global reference frame. The kinematic model of R is given by Equation 1:

$${\dot{q}}_{R}\left(t\right)=\left[\begin{array}{c} {\dot{x}}_{R}\left(t\right)\\ {\dot{y}}_{R}\left(t\right)\\ {\dot{\theta }}_{R}\left(t\right) \end{array}\right]=\left[\begin{array}{c} v_{R}\left(t\right)\cos\theta_{R}\left(t\right)\\ v_{R}\left(t\right)\sin\theta_{R}\left(t\right)\\ \omega_{R}\left(t\right) \end{array}\right]$$ (1)

where $v_{R}\left(t\right)$ and $\omega_{R}\left(t\right)$ correspond to the linear and angular velocities of the mobile robot, respectively. The robot state can be estimated by dead reckoning by integrating the kinematic model over time: $q_{R}\left(t\right)=q_{R}\left(t_{0}\right)+\int_{t_{0}}^{t}{\dot{q}}_{R}\left(\tau \right)\;d\tau$ .

Let M be the robotic manipulator representing the LIAC HWL. The accelerator is modeled as a mobile manipulator composed of a robotic arm mounted on a mobile base.

Let’s assume that the manipulator has n degrees of freedom with joint configuration represented by $q_{M}\left(t\right)={\left[q_{1}\left(t\right)\;\;q_{2}\left(t\right)\;\ldots \;q_{n}\left(t\right)\right]}^{T}$ . The pose of the manipulator end-effector with respect to the global reference frame (world) is obtained through the composition of the mobile base transformation and the manipulator forward kinematics TEW(qM)=TBW⋅TEB(qM) , where W , B and E denote the world, base and end-effector frames, respectively. The transformation of the mobile base is expressed as TBW = RBWtBW01∈SE(3) , where RBW represents the rotation matrix and tBW the translation vector of the mobile base with respect to the global reference frame. The forward kinematics of the manipulator is defined as TEB(qM)=∏i=1nTii−1(qi) , where Tii−1(qi) denotes the homogeneous transformation associated with the i -th joint following the standard homogeneous-transformation formulation for serial manipulators (Siciliano et al., 2009). Without loss of generality, it can be assumed that, for a specific configuration of the LIAC HWL, the base of the manipulator is static and its pose (position and orientation) is known.

Let’s define the Euclidean distance between the target point and the position of the mobile robot as follows:

$$e\left(t\right)=\|p_{T}-p_{R}\left(t\right){\|}_{2}=\sqrt{{\left(x_{T}-x_{R}\left(t\right)\right)}^{2}+{\left(y_{T}-y_{R}\left(t\right)\right)}^{2}}$$ (2)

where $p_{T}\in R^{2}$ is the target position and $p_{R}\left(t\right)\in R^{2}$ corresponds to the planar position extracted from the complete robot state $q_{R}\left(t\right)$ .

Therefore, the objective of the positioning task is to minimize the beam stopper error at the final time instant $t_{\mathrm{end}}$ , as expressed in Equation 3:

$$\underset{u_{R}\left(t\right)}{\min}e\left(t_{\mathrm{end}}\right)$$ (3)

where $u_{R}\left(t\right)={\left[v_{R}\left(t\right)\;\;\omega_{R}\left(t\right)\right]}^{T}$ represents the control input of the mobile platform over time, which is bounded by the physical limitations of the differential platform such that $|v_{R}\left(t\right)|\leq v_{\max}$ and $|\omega_{R}\left(t\right)|\leq \omega_{\max}$ .

## 3 Experimental methodology

This section describes the experimental methodology adopted to evaluate the proposed beam stopper positioning system. First, the experimental setup and the robotic platforms involved in the study are presented. Next, the audiovisual assistance system developed to support the positioning task is described. Finally, the experimental study designed to assess the performance of the proposed approach is detailed.

### 3.1 Experimental setup

To evaluate the proposed system under controlled conditions, an experimental environment has been designed that reproduces the basic spatial arrangement of an IOERT procedure. The setup includes the two main robotic platforms presented in Section 2.

The robotic manipulator allows the reproduction of different accelerator head orientations equivalent to pitch and roll angular variations during treatment delivery. In parallel, the mobile robot operates beneath a table that simulates the surgical operating table. This configuration enables the study of beam stopper positioning in different spatial beam configurations. Figure 2 illustrates the similarity between a real clinical procedure and the experimental environment proposed.

**FIGURE 2:**
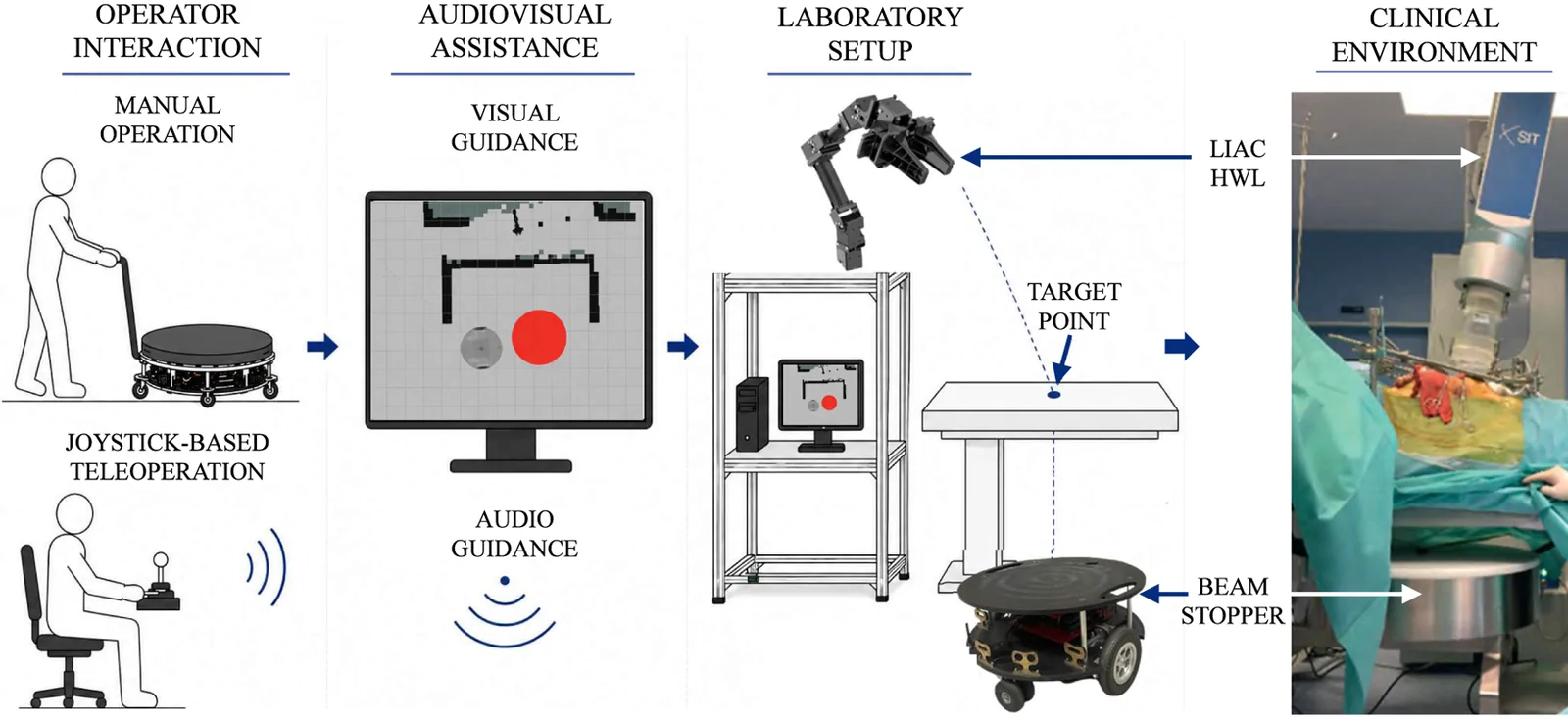
Experimental setup for IOERT procedure.

The robotic manipulator M used in the experiments is the OpenManipulator-X (RM-X52-TNM) manufactured by ROBOTIS (ROBOTIS Co. Ltd, 2017). The platform consists of four rotational degrees of freedom together with a gripper-type end-effector providing a fifth degree of freedom. Each joint is actuated using intelligent servomotors from the DYNAMIXEL family, specifically the XM430-W350-T model, integrating position and velocity control capabilities. Low-level control was implemented through an OpenCR (Open-source Control module for ROS) controller, while high-level processing tasks were executed on a Raspberry Pi 5 platform.

For this particular implementation, the mobile robot R is the Eddie platform developed by Parallax Inc (Parallax Inc, 2013) with a differential-drive kinematic configuration, including two motorized wheels and two passive caster wheels positioned at the front and rear sections to provide stability. The power and control electronics has been customized for full integration within Robot Operating System 2 (ROS 2), including wheel encoders and inertial measurement unit (IMU) for odometry estimation with an Extended Kalman Filter (EKF). For low-level control an ESP32 microcontroller is used. The robotic platform also includes a rotary laser range sensor (LiDAR), model RPLIDAR A2 from SLAMTEC, used for localization and mapping. For high-level processing a Raspberry Pi 5 platform runs a ROS 2 Jazzy distribution and communicates with the ESP32 through MicroROS.

The complete system followed a centralized communication architecture, as shown in Figure 3, where a central computer received information from both robotic platforms and processed the data required to estimate the target point and generate the audiovisual assistance system. The mobile robot continuously transmitted odometry and LiDAR data via Wi-Fi, while the manipulator transmitted the angular measurements of its joints. Using this information, the central computer estimated the manipulator pose and orientation and computed the point where the radiation beam intersected the floor, which defined the target position for the beam stopper.

**FIGURE 3:**
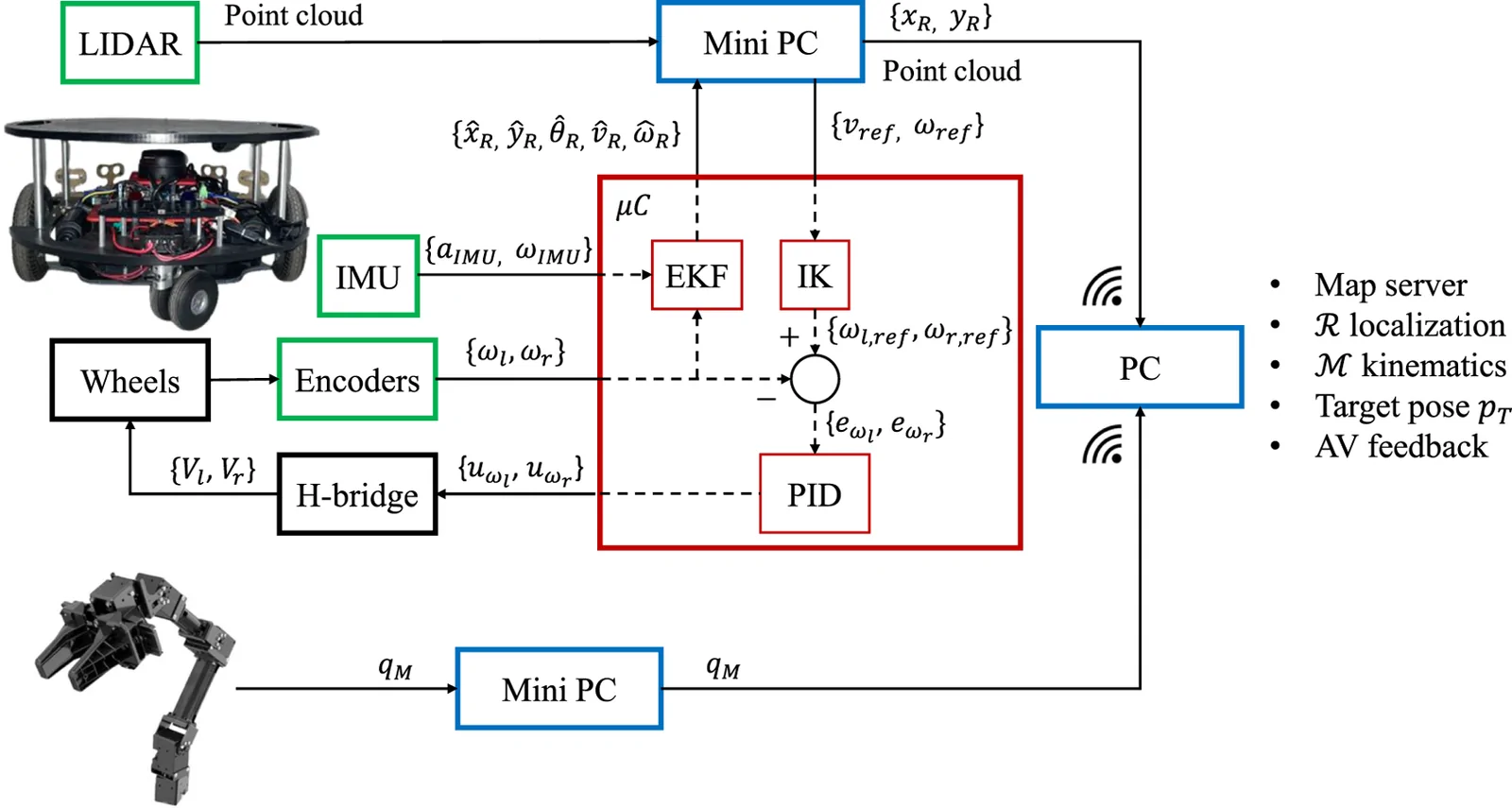
Communication architecture for the experimental setup. Green boxes denote sensing modules (LiDAR, IMU and wheel encoders), blue boxes denote the high-level computing units (Mini PCs and central PC), the red block denotes the embedded low-level control subsystem, including the microcontroller ( μ C), Extended Kalman Filter (EKF), inverse kinematics (IK) and proportional–integral–derivative (PID) controller, and black boxes denote the actuation/power hardware (wheels and H-bridge). The mobile robot R transmits localization and LiDAR data to the central PC via Wi-Fi, while the manipulator M transmits its joint configuration $q_{M}$ . The central PC performs map-server functions, estimates the target pose $p_{T}$ and generates the audiovisual (AV) feedback. LiDAR: Light Detection and Ranging; IMU: inertial measurement unit; PC: personal computer.

To estimate this target point, the radiation beam was modeled as a three-dimensional line whose origin is the end-effector position and whose direction is defined by the end-effector orientation. Let tEW denote the beam emission point in the world frame. Assuming that the beam propagates along the local z -axis of the end-effector, the beam direction vector is defined as d=REW⋅[001]T . The beam trajectory can therefore be expressed parametrically as r(λ)=tEW+λd , where λ∈R .

The target location was obtained by computing the intersection between this beam trajectory and the ground plane. In the proposed setup, the operating-room floor was assumed to be planar and horizontal, corresponding to the plane z=0 in the map coordinate frame, with normal vector $n={\left[0\;\;0\;\;1\right]}^{T}$ . A plane in three-dimensional space is defined by $n^{T}\left(x-p_{0}\right)=0$ , where $p_{0}$ is any point belonging to the plane. Substituting the beam equation into the plane equation yields nT(tEW+λd−p0)=0 , from which the parameter $\lambda^{*}$ can be obtained using Equation 4:

λ*=nTp0−tEWnTd (4)

Finally, the beam-floor intersection point is computed as pT=tEW+λ*d . This point represents the target point that the beam stopper has to reach and it is used to calculate the distance described in Equation 2.

### 3.2 Proposed audiovisual assistance system

As described in the beginning, beam stopper positioning is currently performed without a verification system capable of providing a direct reference to ensure accurate alignment between the beam stopper and the radiation beam. To address this limitation, an audiovisual assistance system has been implemented.

For the visual feedback interface for the operator, RViz has been employed. Based on the estimated relative position between the target point and the current beam stopper position, a dynamic graphical representation has been generated to provide intuitive guidance during the positioning task.

The visual assistance consists of a dynamic target composed of concentric circles progressively displayed as the positioning error e(t) decreases. To generate this behavior, several positioning-error thresholds are defined according to the following conditions:

- if e(t)>1.00 m , no ring is displayed;
- if 0.40 m<e(t)≤1.00 m , the outer ring is displayed;
- if 0.20 m<e(t)≤0.40 m , two rings are displayed;
- if 0.05 m<e(t)≤0.20 m , three rings are displayed;
- and if e(t)≤0.05 m , all rings are displayed, indicating maximum proximity to the target point.

The positioning-error thresholds were empirical interface-design choices introduced to provide progressively finer guidance as the operator approached the target. The outer threshold of 1.00 m was selected to define an initial proximity zone slightly larger than the approximate 0.70 m diameter of the clinical beam stopper, so that the interface clearly indicated when the robot had entered the target vicinity. The intermediate thresholds of 0.40 and 0.20 m were then introduced to provide progressively finer visual and auditory guidance during the approach. These values were not derived from published clinical positioning criteria and, to the best of the authors’ knowledge, no published reference establishes these specific thresholds for beam-stopper positioning. The innermost threshold of 0.05 m was defined as the experimental criterion for successful positioning. Its selection was based on the physical dimensions of the clinical beam stopper and on consultation with a domain expert with direct experience using the LIAC HWL system. The approximate beam-stopper diameter of 0.70 m was communicated by this expert. This information is not specified in the publicly available LIAC HWL technical documentation and, to the best of the authors’ knowledge, is not reported in a published source. Therefore, the 0.05 m value should be interpreted as an expert-informed and deliberately stringent experimental centering criterion rather than as a published clinical safety tolerance.

The visual markers are continuously updated in real time using the information published by the robot control system. Figure 4 shows the visual assistance interface in RViz while the concentric circles progressively appear as the mobile robot approaches the target point. This approach provides an intuitive and easily interpretable representation of the positioning accuracy achieved during the task.

**FIGURE 4:**
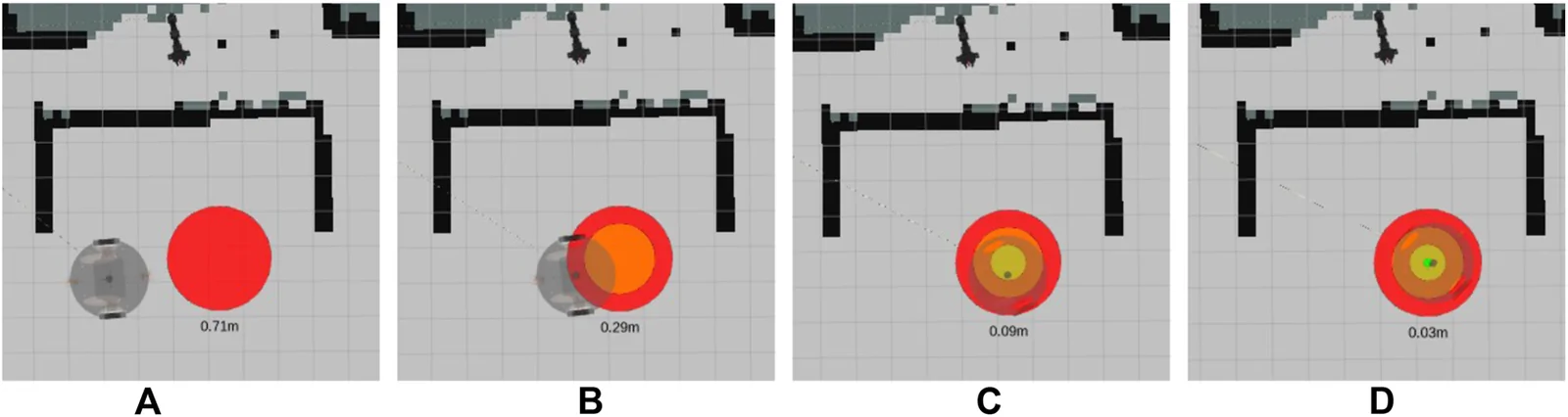
Visual assistance interface showing the dynamic target thresholds: **(A)** 0.40<e(t)≤1.00 m, **(B)** 0.20<e(t)≤0.40 m, **(C)** 0.05<e(t)≤0.20 m, and **(D)** e(t)≤0.05 m.

In addition to the visual interface, an auditory assistance system based on discrete acoustic signals has been implemented. The frequency of the acoustic signals increased as the distance to the target point decreases. This mechanism follows a principle analogous to that used in parking assistance systems, where the repetition rate of the acoustic warnings is inversely proportional to the distance to the obstacle (Niewels et al., 2013).

In the proposed system, the beep frequency has been parameterized as a function of the positioning error e(t) and follows the same positioning-error threshold defined for the visual feedback. Consequently, the auditory assistance provides an additional perceptual reference that does not require continuous visual attention, facilitating spatial alignment during both manual and teleoperated positioning tasks.

### 3.3 Experimental study

The experimental study was conducted with a previously selected group of participants (N=20) and compares four different operating modalities varying according to the transportation method of the mobile robot and the use or absence of the audiovisual assistance system. The sample comprised 12 male and 8 female participants. Regarding age, 9 participants were aged 18–34 years (45%), 7 were aged 35–54 years (35%), and 4 were aged 55 years or older (20%). All participants belonged to the academic community, including 9 engineering students and 11 researchers and professors. Participants were recruited through a voluntary invitation within the academic environment; participation was entirely voluntary and no financial or other compensation was provided. None were recruited on the basis of professional experience in radiation oncology or medical physics. Consequently, the sample should be regarded as a non-clinical convenience sample with a technical/engineering background rather than as representative of an IOERT clinical team.

Regarding the transportation method, two operating conditions are defined: manual and teleoperated. In the manual mode, participants are required to move the mobile robot by physically pushing or pulling it toward the target point. This procedure reproduces the positioning method currently performed in oncological operating rooms during IOERT procedures. In contrast, in the teleoperated mode, participants control the mobile robot remotely using a joystick interface. This configuration introduces a partial degree of automation in the positioning process in order to analyze whether such modification could improve positioning accuracy and ease of operation compared with manual transportation.

When both modalities are performed without audiovisual assistance, participants are required to estimate the target position exclusively by observing the orientation of the robotic manipulator and inferring the location where the beam intersected the floor plane, similarly to the procedure currently followed in clinical operating rooms. Conversely, when audiovisual assistance is available, participants can still estimate the target position visually while additionally relying on the audiovisual information provided by the proposed assistance system.

Consequently, four experimental operating modalities are evaluated: manual mode without assistance, manual mode with assistance, teleoperated mode without assistance and teleoperated mode with assistance. This classification allows the independent evaluation of both the transportation method and the contribution of the audiovisual assistance system to the overall positioning performance.

To conduct the experiments, a set of predefined target points have been distributed around the table simulating the surgical operating table. These points have been grouped into four different spatial regions representing distinct manipulator configurations depending on the incidence angle of the simulated radiation beam, either beneath the table or in positions external to it. Figure 5 illustrates the different experimental regions. Region 1 corresponds to a fully occluded area beneath the table, Region 2 represents a partially occluded area and Regions 3 and 4 correspond to non-occluded lateral areas located on the right and left sides of the table, respectively. The four regions are an experimental classification defined by the authors according to the degree of visual occlusion and the relative table–target geometry; they are not intended to represent medically defined anatomical or dosimetric regions. Their purpose is to create reproducible spatial conditions with different levels of perceptual difficulty for the positioning task.

**FIGURE 5:**
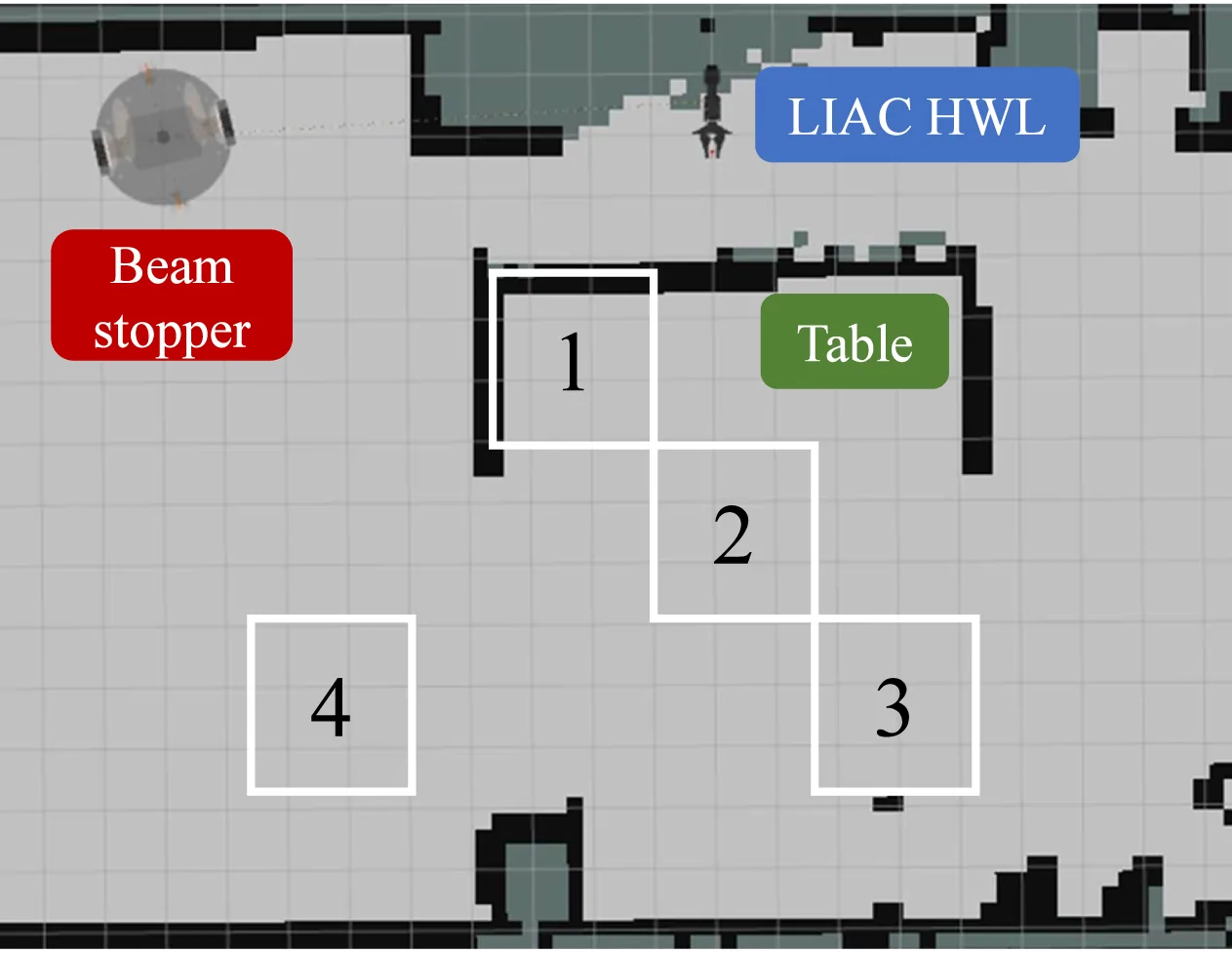
Experimental regions around the simulated operating table. Z1: fully occluded region beneath the table; Z2: partially occluded region; Z3 and Z4: non-occluded lateral regions on the right and left sides, respectively. The beam stopper is represented by the mobile robot and the table footprint is shown at the center.

This spatial distribution enables the analysis of positioning difficulty and accuracy as a function of the degree of occlusion imposed by the operating table. Four equidistant target points have been defined within each region to guarantee homogeneous evaluation across all experimental conditions. The selection and distribution of the target points are additionally intended to reduce the effect of participant learning throughout the experimental sessions.

Since two of the evaluated modalities incorporate audiovisual assistance, there exists the possibility that participants could memorize target locations and consequently bias the experimental results. To mitigate this effect, a total of 16 target points has been considered and randomly distributed among the four experimental modalities, assigning four target points to each operating condition.

Furthermore, the execution sequence has been generated according to several restrictions:

1. The same operating mode cannot be repeated more than twice consecutively;
2. The four target points assigned to each mode belong to different spatial regions;
3. Each participant performs the experiments following an individualized randomized order;
4. The first four trials correspond randomly to the four operating modalities.

The experimental evaluation primarily considers two performance metrics: positioning accuracy and task completion time. Positioning accuracy is quantified using the positioning error e(t) defined in (Equation 2), considering a successful positioning whenever e(t)≤0.05 m. Task timing was controlled by the experimenter. At the beginning of each trial, the participant verbally indicated readiness to start and the experimenter initiated the acquisition. When the participant considered the mobile robot to be in its final position, the participant stated this verbally; the experimenter then stopped the trial and recorded the final robot pose. Thus, confirmation did not require the participant to press a button, operate an additional interface or change position, minimizing interference of the confirmation procedure with the positioning task.

In addition to the objective evaluation metrics, two post-experimental electronic questionnaires were used to complement the analysis with subjective user-experience information. Both questionnaires were completed immediately after the participant had finished the full set of experimental trials, during the same experimental session rather than at a later date. The first questionnaire consists of several sections intended to characterize the participant profile and their perception during the experiments, while the second questionnaire is based on the NASA-TLX methodology (Said et al., 2020).

## 4 Results

### 4.1 Effect of interaction mode and audiovisual assistance

To evaluate the impact of audiovisual assistance and interaction mode on task performance, both the final positioning error and the time required to complete the task have been analyzed across the four experimental conditions considered. Table 1 summarizes the mean values and standard deviations obtained for each operating mode.

**TABLE 1:** Mean and standard deviation of positioning error and execution time for the four experimental conditions.

Condition	$\bar{e}\pm \sigma_{e}$ [m]	$\bar{t}\pm \sigma_{t}$ [s]
Manual	0.3077±0.1902	53.48±22.17
Manual + assistance	0.0497±0.0342	56.01±29.78
Teleoperation	0.2914±0.2271	66.80±33.96
Teleoperation + assistance	0.0632±0.0425	64.85±37.81

The results demonstrate that audiovisual assistance has a much stronger impact on positioning accuracy than the interaction modality itself. As shown in Table 1, while manual and teleoperated operation achieve comparable performance without assistance, the incorporation of audiovisual guidance leads to a substantial reduction in positioning error in both cases, with improvements of approximately 80%. Furthermore, the assisted conditions exhibit lower inter-participant variability, indicating more consistent task execution.

Figure 6A illustrates the distribution of the final positioning error for the four evaluated operating modes. In the non-assisted conditions, a considerable dispersion of errors can be observed, including several trials with positioning errors close to 1 m and one outlier exceeding 1.5 m. In contrast, under the assisted conditions, most positioning errors remain below 0.1 m and exhibit substantially more compact distributions.

**FIGURE 6:**
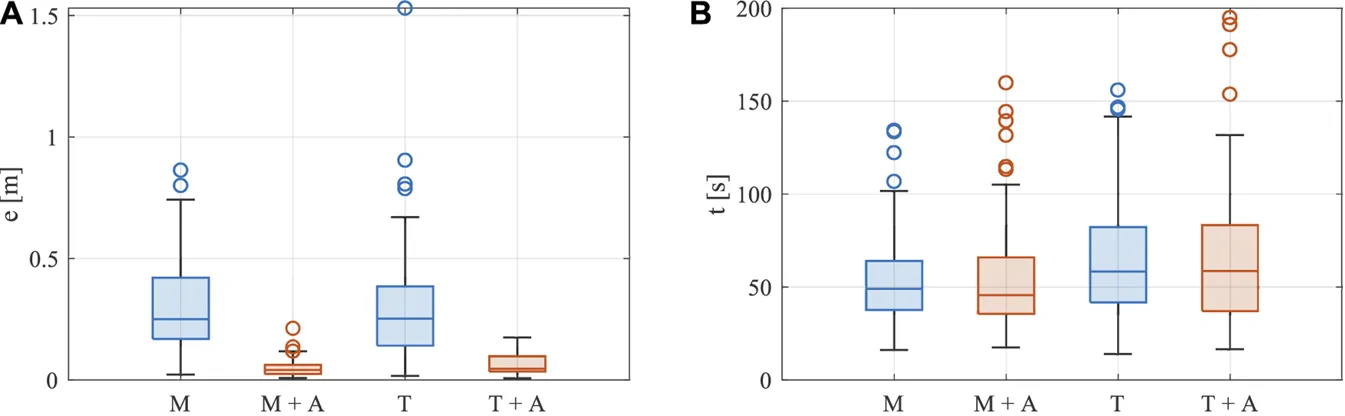
Distribution of positioning performance across the four experimental conditions: M (manual operation); M + A (manual operation with audiovisual assistance); T (teleoperated operation); T + A (teleoperated operation with audiovisual assistance). **(A)** Final positioning error. **(B)** Task completion time.

Regarding the time required to complete the task, the differences between conditions are less pronounced. As shown in Table 1, the manual mode exhibits the shortest mean execution time, followed by the assisted manual mode. In contrast, the teleoperated modes require longer completion times, with the non-assisted teleoperation condition showing the highest mean execution time $\bar{t}\approx 67$ s.

Figure 6B presents the distribution of task completion times for the four experimental conditions. Unlike the positioning error results, execution times exhibit considerable variability across all operating modes, with broad distributions and several particularly long trials. Nevertheless, the median values for the teleoperated modes are consistently higher than those observed for manual positioning, reflecting the greater complexity associated with remote robot control.

These observations are supported by the two-way repeated-measures analysis of variance (ANOVA) (Field, 2024). The analysis confirms that audiovisual assistance has a highly significant effect on positioning accuracy $\left(p=6.44\times 10^{-10}\right)$ , whereas the interaction mode does not significantly affect the final positioning error (p=0.933) . In contrast, the interaction mode significantly influences the completion time of the task (p=0.013) , teleoperation requiring longer execution times than manual operation, while audiovisual assistance does not affect the duration of the task (p=0.997) . No significant interaction between both factors is found for either positioning error (p=0.308) or execution time (p=0.570) , indicating that the benefit provided by audiovisual assistance is consistent across both interaction modalities. To complement statistical significance with an estimate of practical magnitude, partial eta-squared $\left(\eta_{p}^{2}\right)$ is also reported. For final positioning error, the effect sizes were $\eta_{p}^{2}\approx 0.872$ for audiovisual assistance, $\eta_{p}^{2}\approx 0.0004$ for interaction mode, and $\eta_{p}^{2}\approx 0.055$ for their interaction. For task completion time, the corresponding values were $\eta_{p}^{2}\approx 0.283$ for interaction mode, $\eta_{p}^{2}<0.001$ for audiovisual assistance, and $\eta_{p}^{2}\approx 0.017$ for the interaction. These values confirm that the dominant practical effect is the contribution of audiovisual assistance to positioning accuracy, whereas interaction mode has a comparatively larger effect on completion time.

### 4.2 Effect of workspace region

After evaluating the effects of audiovisual assistance and interaction mode on task performance, the influence of target location within the environment has been analyzed. Specifically, the final positioning error and task execution time are examined across the different workspace regions (Z1–Z4).

The analysis is first conducted globally by grouping the experimental conditions according to the presence or absence of audiovisual assistance. Subsequently, each interaction mode is analyzed independently. Finally, the statistical influence of workspace region is assessed through repeated-measures ANOVA.

With regard to the influence of workspace region as a function of audiovisual assistance, Figure 7 presents the boxplots of positioning error and task execution time for each workspace region.

**FIGURE 7:**
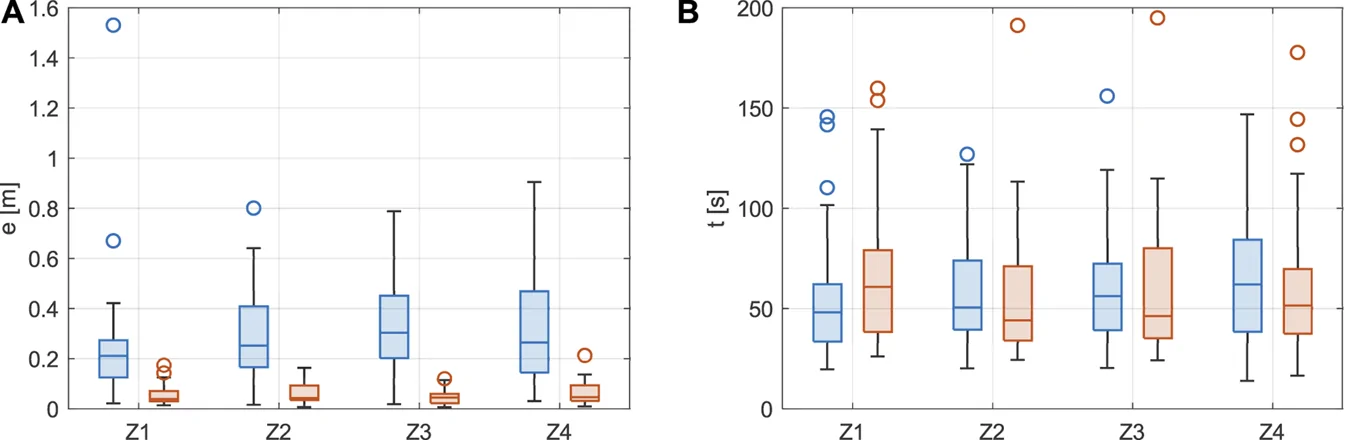
Effect of workspace region on positioning performance across regions (Z1–Z4), grouped according to the absence (blue) and presence (red) of audiovisual assistance. **(A)** Final positioning error. **(B)** Task completion time.

As shown in Figure 7A, the distributions corresponding to the assisted condition exhibit lower median values and narrower interquartile ranges than those observed in the non-assisted condition across all regions. Under the non-assisted condition, Z1 presents the lowest median positioning error despite the presence of several outliers. In contrast, when audiovisual assistance is available, all four regions exhibit similar median values. This trend is also reflected in the mean values calculated for each region.

For the non-assisted condition, Z1 yields a mean positioning error of $\bar{e}=0.24$ m with a standard deviation of $\sigma_{e}=0.24$ m, whereas the remaining regions exhibit mean errors of approximately $\bar{e}=0.32$ m, with standard deviations of $\sigma_{e}=0.17$ m for Z2, $\sigma_{e}=0.18$ m for Z3, and $\sigma_{e}=0.22$ m for Z4. Conversely, under the assisted condition, all regions show mean positioning errors of approximately $\bar{e}=0.05$ m and standard deviations close to $\sigma_{e}=0.04$ m.

Regarding task execution time, Figure 7B indicates that the median values are generally comparable across conditions, although considerable dispersion and the presence of outliers can be observed in several regions. Under the non-assisted condition, Z4 exhibits the highest median execution time, whereas under the assisted condition, the highest median is observed in Z1.

The mean values reveal a similar trend. In the non-assisted condition, Z4 presents the longest average execution time, with a mean of $\bar{t}=67$ s and a standard deviation of $\sigma_{t}=36$ s. The remaining regions exhibit standard deviations of approximately $\sigma_{t}=28$ s. In the assisted condition, Z1 shows the highest mean execution time, reaching $\bar{t}=65$ s with a standard deviation of $\sigma_{t}=34$ s. Although Z1 records the highest mean value, Z4 remains the region with the greatest variability, exhibiting a standard deviation of $\sigma_{t}=37$ s.

The influence of workspace region has also been analyzed separately for each of the four experimental operating modes. Figure 8 presents the distribution of the final positioning error across workspace regions for each operating mode.

**FIGURE 8:**
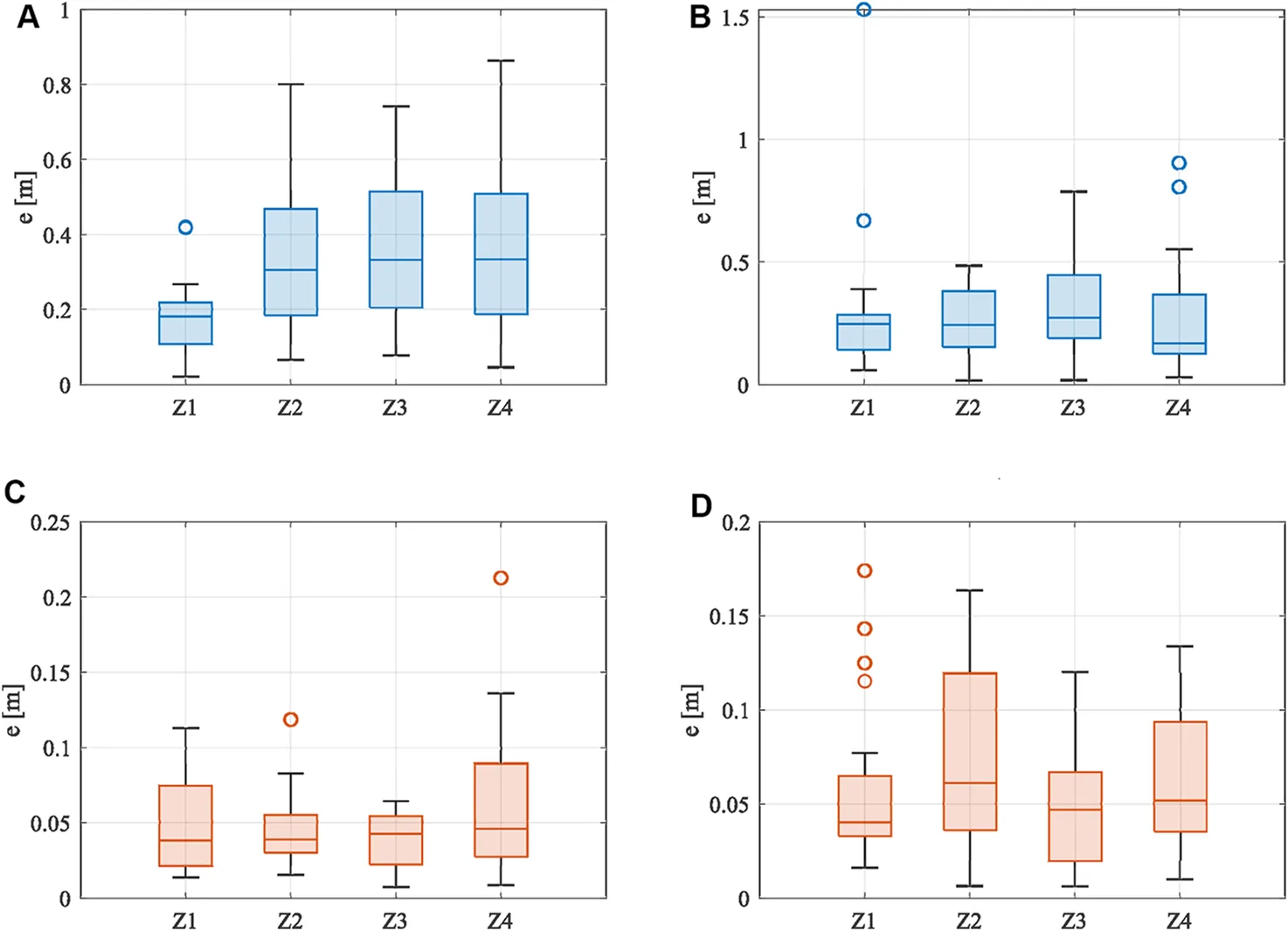
Boxplots of final positioning error across workspace regions (Z1–Z4) for the operating modes. **(A)** Manual. **(B)** Teleoperation. **(C)** Manual + Assistance. **(D)** Teleoperation + Assistance.

The non-assisted manual condition exhibits the largest regional differences, with Z1 achieving the lowest positioning errors, whereas the remaining regions show higher mean errors and greater variability. In contrast, the non-assisted teleoperation condition presents more homogeneous positioning errors across the workspace, although with a generally larger dispersion.

The introduction of audiovisual assistance substantially improves positioning accuracy in both interaction modalities. Under both assisted manual and assisted teleoperation conditions, positioning errors remain consistently low across all workspace regions, with similar median values and limited variability. These results indicate that the performance gains provided by audiovisual assistance are maintained regardless of target location within the workspace.

Although execution time shows greater variability than positioning error, no clear regional trends are observed for any of the four operating modes. Minor differences between regions are limited to isolated increases in variability and do not translate into consistent changes in task duration.

These trends are further confirmed by the repeated-measures ANOVA. When the experimental conditions are grouped according to the availability of audiovisual assistance, workspace region shows only a trend toward significance for positioning error in the non-assisted condition (p=0.092) , whereas no significant regional effect is observed with audiovisual assistance (p=0.778) . Likewise, workspace region does not significantly affect task completion time either without (p=0.190) or with assistance (p=0.570) .

When each interaction mode is analyzed separately, a significant effect of workspace region is found only in the non-assisted manual condition (p=0.002) . This effect disappears once audiovisual assistance is incorporated, with only a marginal trend remaining in the assisted manual condition (p=0.052) , while no significant regional differences are observed for either teleoperation condition. Furthermore, workspace region does not significantly influence task completion time in any of the evaluated operating modes. Overall, these findings indicate that audiovisual assistance effectively mitigates the influence of target location on positioning accuracy while preserving homogeneous temporal performance throughout the workspace.

### 4.3 Effect of manipulator geometry

To further investigate the origin of the spatial performance differences observed in the previous analyses, the relationship between positioning error and the geometric configuration of the manipulator with respect to the environment has been examined. Two geometric parameters are considered: the angle of the manipulator with respect to the vertical, denoted by δ , and the horizontal distance between the manipulator and the target location, denoted by L .

The vertical angle δ characterizes the orientation of the simulated radiation-beam axis with respect to the ground surface. Considering the ground plane normal vector n and the beam direction vector d , the angle between the beam and the plane normal can be computed using Equation 5:

$$\delta =\arccos\left(\left|d^{T}n\right|\right)$$ (5)

where δ represents the angle between the beam direction and the normal to the ground plane. Small values of δ correspond to configurations in which the beam is nearly perpendicular to the ground surface, whereas larger values indicate increasingly oblique beam orientations.

In addition, the horizontal distance between the position of manipulator end-effector $\left(p_{E}={\left[x_{E}\;\;y_{E}\right]}^{T}\right)$ and the target location $\left(p_{T}={\left[x_{T}\;\;y_{T}\right]}^{T}\right)$ has been analyzed. This parameter quantifies the lateral displacement between the vertical projection of the end-effector and the beam impact point on the ground. The horizontal distance L is computed using Equation 6:

$$L=\sqrt{{\left(x_{T}-x_{E}\right)}^{2}+{\left(y_{T}-y_{E}\right)}^{2}}$$ (6)

Larger values of L indicate configurations in which the target location is farther from the manipulator base.

Figure 9 presents the boxplots of positioning error obtained for each experimental point (P1–P4) across the four workspace regions. In each subfigure, the blue boxplots correspond to the non-assisted condition, whereas the red boxplots represent the condition with audiovisual assistance. Overall, audiovisual assistance reduces both the magnitude and the variability of the positioning error across all evaluated experimental points.

**FIGURE 9:**
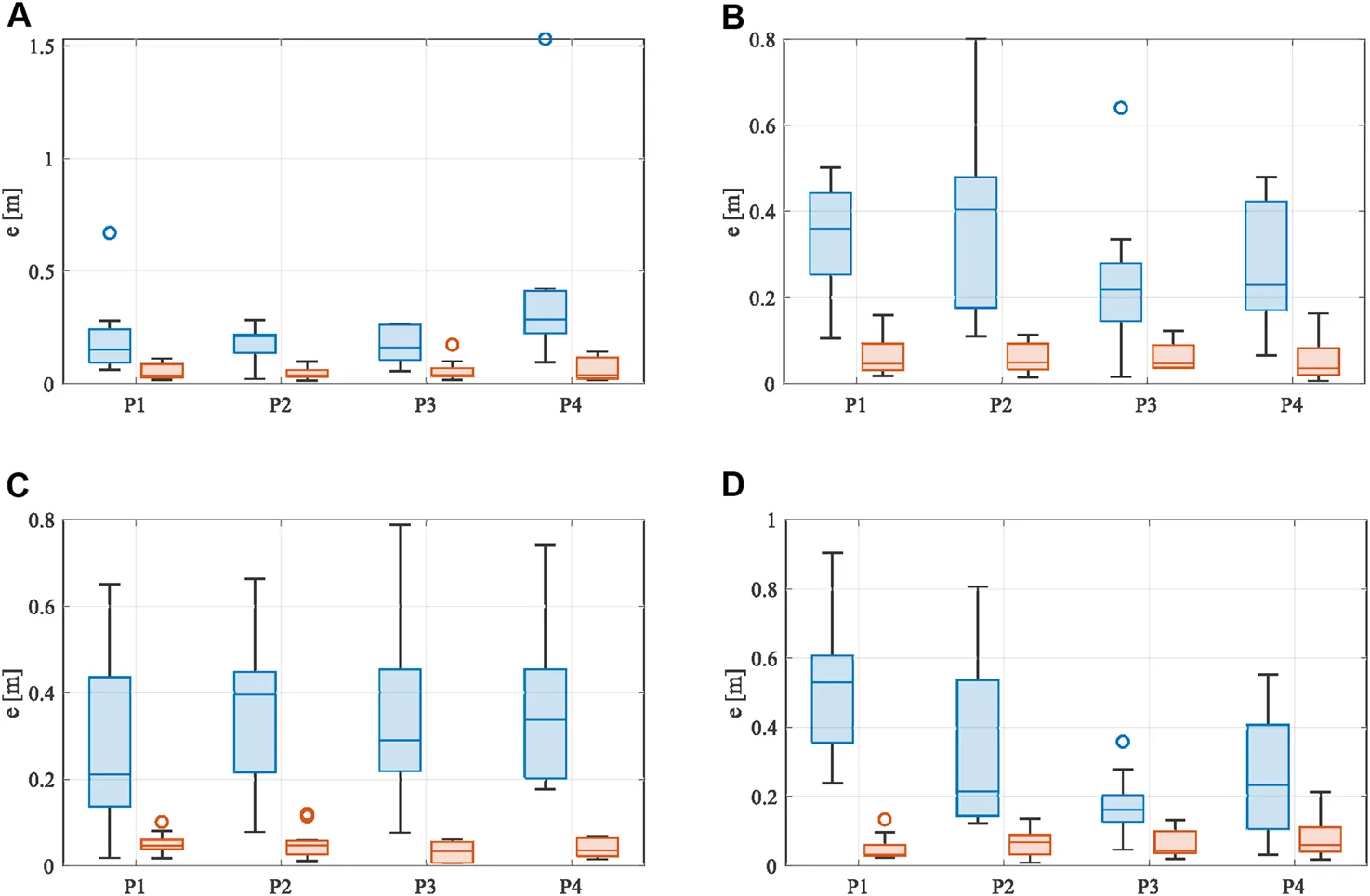
Distribution of final positioning error at each experimental point (P1–P4) for the four workspace regions under non-assisted (blue) and audiovisual assistance (red) conditions. **(A)** Region Z1. **(B)** Region Z2. **(C)** Region Z3. **(D)** Region Z4.

In Z1 (see Figure 9A), the non-assisted condition exhibits a clear dependence on the experimental point. P2 and P3 show the most compact distributions and the lowest median errors, whereas P4 exhibits the largest positioning errors and the highest variability. This trend is consistent with the corresponding mean values, with the lowest errors observed for P2 ( $\bar{e}=0.18$ m) and P3 ( $\bar{e}=0.17$ m), while P4 reaches a mean positioning error of $\bar{e}=0.40$ m. The presence of outliers further suggests that certain participants experienced additional difficulties under specific spatial configurations.

A similar trend can be observed in Z2 (see Figure 9B), where P2 exhibits both the largest dispersion and the highest positioning errors. This is also reflected in the mean values, with a mean error of $\bar{e}=0.63$ m recorded for this configuration. In contrast, the remaining experimental points exhibit lower errors and reduced variability. Likewise, noticeable differences between experimental points can be observed in Z3 (see Figure 9C), although these differences are less pronounced than those found in Z2. Finally, in Z4 (see Figure 9D), P1 stands out as the most challenging configuration, exhibiting the highest median error, a broad distribution and a mean positioning error of $\bar{e}=0.53$ m.

The configurations associated with the largest positioning errors also correspond to the highest manipulator inclination angles and lateral distances. Specifically, P4 in Z1 is characterized by an inclination angle of δ=42.24° and a lateral distance of L=1.21 m, whereas P2 in Z2 reaches δ=50.58° and L=1.52 m. Similarly, P1 in Z4 corresponds to the most demanding geometric configuration, with δ=54.76° and L=1.56 m. These configurations not only exhibit the highest mean positioning errors but also the largest standard deviations, indicating increased sensitivity to inter-participant variability.

These observations provide a geometric explanation for the results previously obtained under the non-assisted condition, where workspace region was found to have a significant effect on final positioning error (p=0.002) . Larger inclination angles combined with greater lateral displacements increase the difficulty of the task, resulting in higher positioning errors and greater variability in performance.

In contrast, the incorporation of audiovisual assistance substantially reduces the differences between experimental points across all four workspace regions. The distributions corresponding to the assisted condition are concentrated near zero, exhibit considerably narrower interquartile ranges and show a substantial reduction in extreme values. This behavior is consistently observed across all subfigures, regardless of the geometric configuration under consideration.

The improvement is particularly evident for the configurations that initially exhibited the highest positioning errors. For example, the mean positioning error at P2 in Z2 decreases from $\bar{e}=0.63$ m to approximately $\bar{e}=0.06$ m, while the mean error at P1 in Z4 is reduced from $\bar{e}=0.53$ m to approximately $\bar{e}=0.05$ m. Similarly, P4 in Z1 shows a substantial reduction in both mean error and variability. Moreover, the differences between experimental points within the same workspace region become almost negligible, suggesting that audiovisual assistance effectively compensates for the difficulties associated with unfavorable manipulator geometries.

### 4.4 User experience analysis

To assess the participants’ subjective perception of the system, a post-experimental questionnaire was administered. The questionnaire was designed to evaluate several aspects related to the user experience with teleoperation systems and audiovisual assistance, including the perceived ease of use of the evaluated operating modes, the perceived usefulness of the assistance system and the perceived difficulty of the task within the experimental environment.

#### 4.4.1 Influence of prior experience

As part of the questionnaire, participants were asked to report their previous experience using joystick-based teleoperation systems. Experience levels were classified into four categories, ranging from 0 (no prior experience) to 3 (high level of experience).

To investigate the influence of prior experience on task performance, the mean positioning error and execution time have been analyzed for each of the four operating modes. Participants were divided into two groups: a low-experience group, comprising users who reported experience levels of 0 or 1, and a high-experience group, comprising users who reported experience levels of 2 or 3. The low-experience group included 11 participants (55%), whereas the high-experience group included 9 participants (45%).

The results indicate that prior joystick experience does not have a statistically significant effect on the final positioning error (p=0.081) . However, a highly significant effect has been found for task execution time $\left(p=5.57\times 10^{-8}\right)$ . Furthermore, a significant interaction between experience level and operating mode has been observed for execution time (p=0.024) , indicating that the influence of experience depends on the interaction mode employed.

Participants with greater joystick experience completed the task substantially faster than those with limited experience, particularly under teleoperated conditions. In the non-assisted teleoperation mode, the mean execution time decreases from $\bar{t}=76.53$ s for the low-experience group to $\bar{t}=54.55$ s for the high-experience group (p=0.002) . A similar effect has been observed in the assisted teleoperation mode, where the mean execution time decreases from $\bar{t}=80.20$ s to $\bar{t}=46.09$ s $\left(p=1.19\times 10^{-5}\right)$ .

In terms of positioning accuracy, the differences between experience groups are considerably smaller. For example, in the non-assisted teleoperation condition, the mean positioning error is $\bar{e}=0.306$ m for low-experience users and $\bar{e}=0.273$ m for high-experience users. Under the assisted teleoperation condition, the corresponding mean errors are $\bar{e}=0.067$ m and $\bar{e}=0.058$ m, respectively. These findings suggest that joystick experience primarily affects operational efficiency and control stability, while having a limited impact on the final positioning accuracy achieved by the user.

#### 4.4.2 Representative operator behavior

To complement the statistical analysis and gain further insight into the differences observed between participants with different experience levels, a detailed temporal analysis of the control and positioning signals has been conducted using two representative participants: one with limited teleoperation experience and one with extensive teleoperation experience. Figures 10–13 illustrate the temporal evolution of the corresponding signals. In all plots, the inexperienced operator is represented in blue, whereas the experienced operator is represented in red.

**FIGURE 10:**
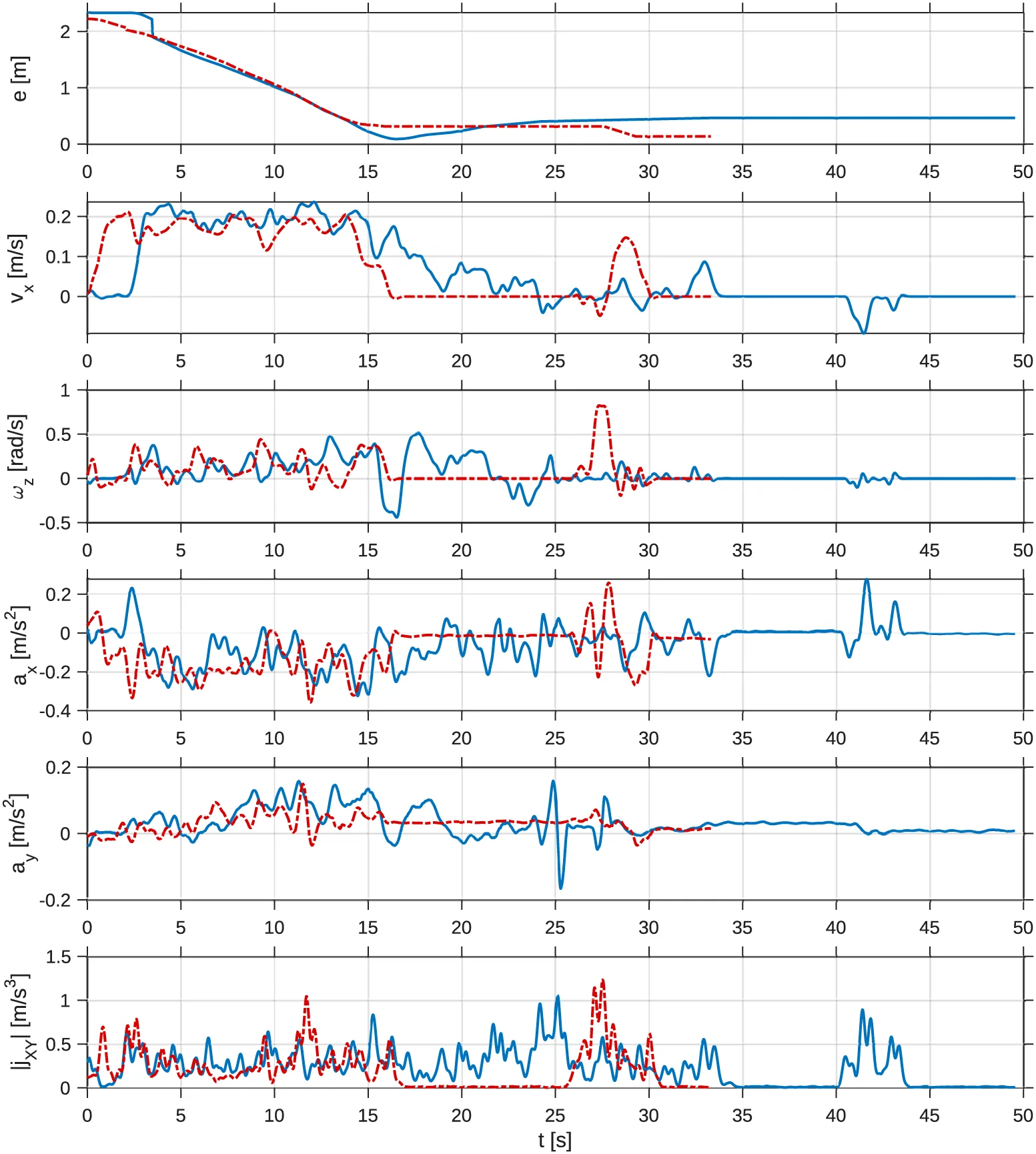
Temporal evolution of the control and positioning variables during the non-assisted manual condition. Inexperienced operator (blue) vs. experienced operator (dashed red).

**FIGURE 11:**
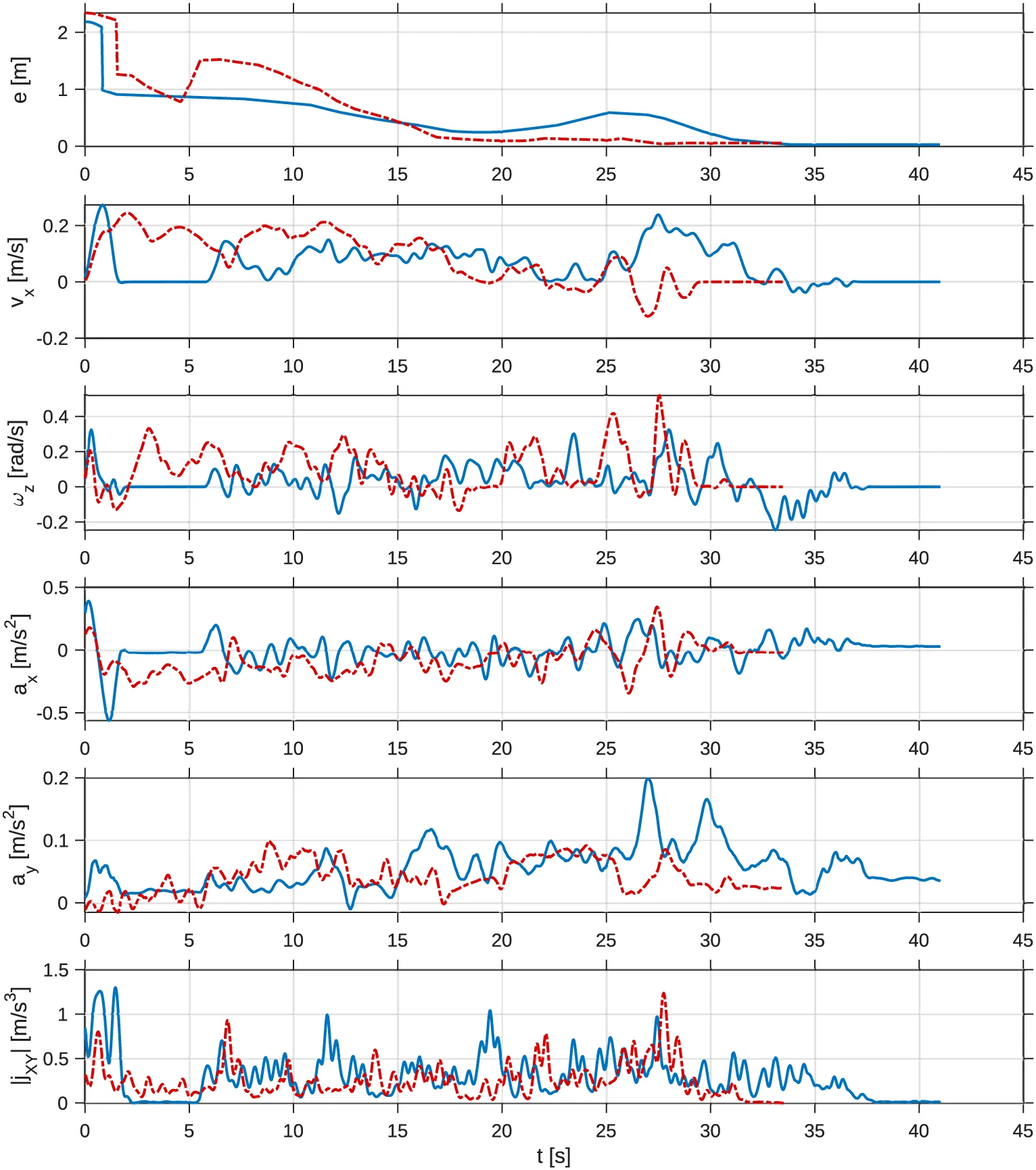
Temporal evolution of control and positioning variables during manual condition with audiovisual assistance. Inexperienced operator (blue) and experienced operator (dashed red).

**FIGURE 12:**
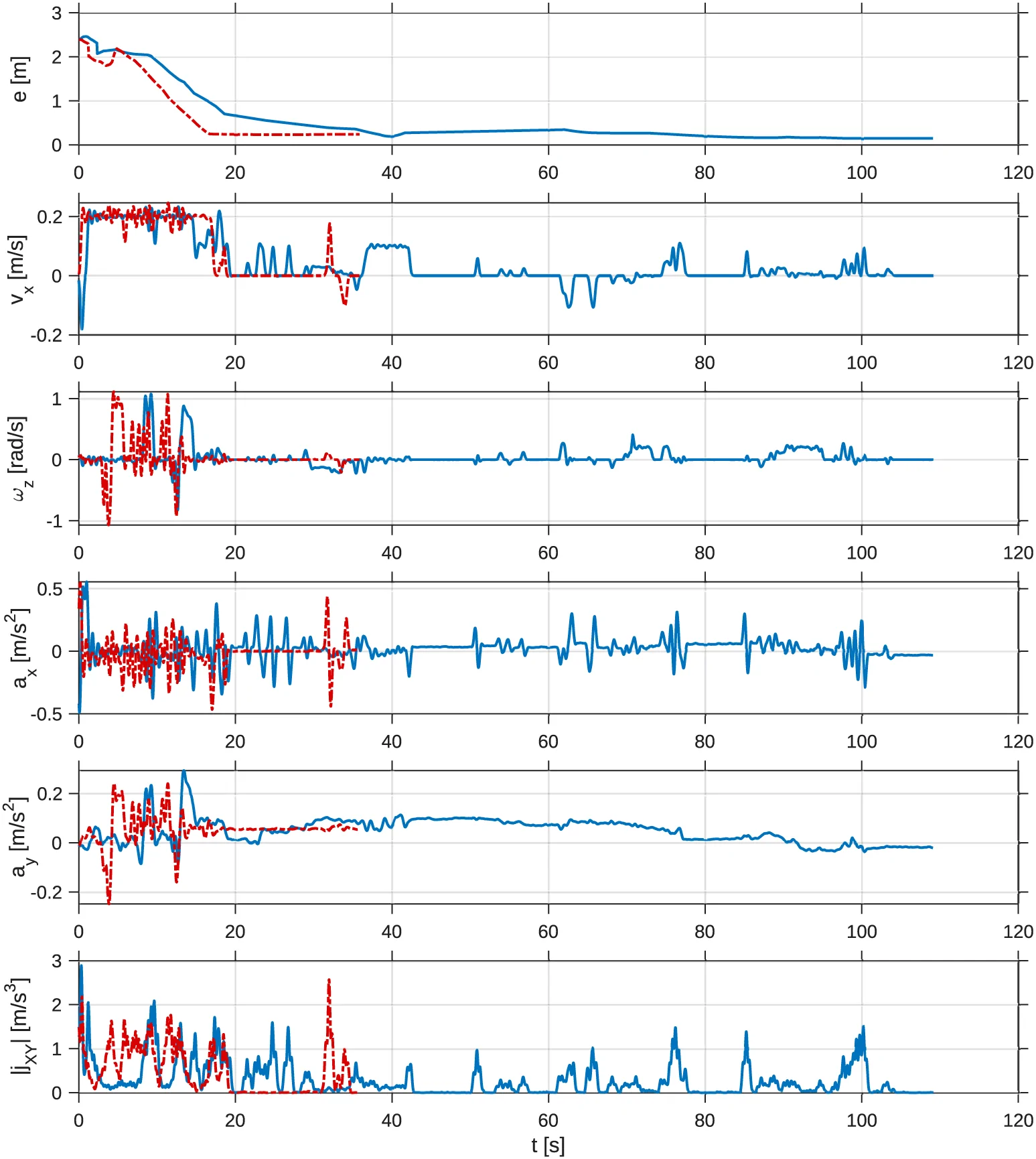
Temporal evolution of control and positioning variables during non-assisted teleoperation condition. Noticeable oscillations occur due to continuous joystick sensitivity adjustments. Inexperienced operator (blue) and experienced operator (dashed red).

**FIGURE 13:**
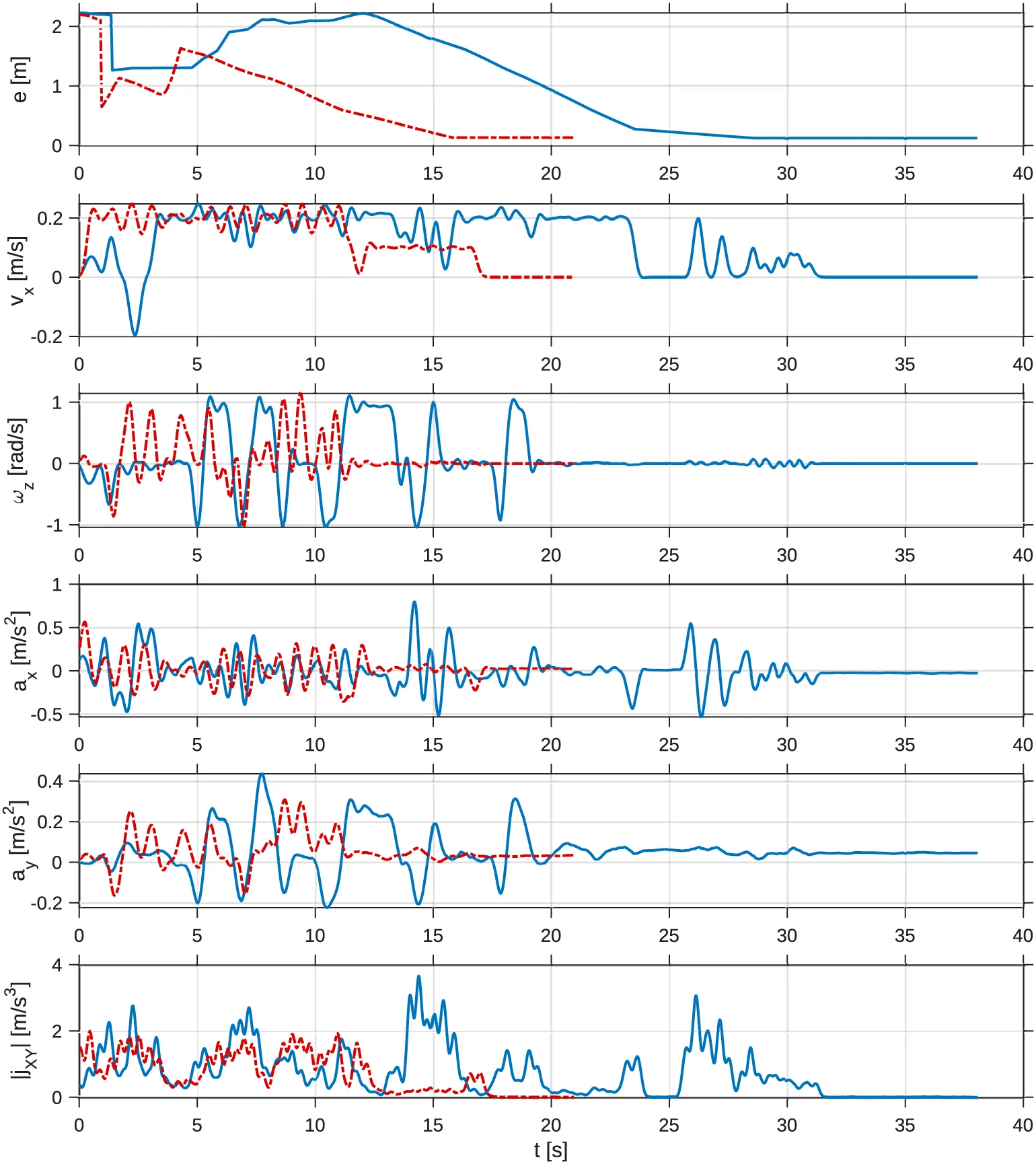
Temporal evolution of control and positioning variables during teleoperation with audiovisual assistance. Inexperienced operator (blue) and experienced operator (dashed red).

Figure 10 presents the behavior of both operators during the non-assisted manual condition. Although both participants ultimately converge to the target position, clear differences can be observed in terms of motion smoothness and control stability.

The experienced operator exhibits substantially smoother and more progressive trajectories, reaching positioning errors e<0.05 m more rapidly, where e is computed as in (Equation 2). In the context of the present task, this error level can already be considered sufficiently accurate for successful task completion. In addition, the experienced participant performs fewer corrective maneuvers during the final approach phase and maintains a more stable evolution of both the positioning error and the linear and angular velocities ( $v_{x}$ and $\omega_{z}$ ) of the mobile robot, which represent the measurement of $v_{R}$ and $\omega_{R}$ , respectively.

By contrast, the inexperienced operator exhibits larger oscillations in both lateral positioning error and robot heading, requiring a considerably greater number of corrective actions before stabilizing the system. These oscillations are accompanied by more abrupt variations in linear and angular velocity, reflecting a less stable control strategy.

The differences become particularly evident during the final approach phase, where the inexperienced operator continues to exhibit residual oscillations even when approaching the desired position. This behavior suggests a reduced ability to perform fine positioning adjustments when task execution relies exclusively on the user’s natural spatial perception.

A similar trend can be observed in the acceleration ( $a_{x}$ , $a_{y}$ ) and jerk $\left(\left|j_{xy}\right|\right)$ profiles. These variables represent the measurement of local acceleration with respect to the longitudinal ( x -axis) and transverse ( y -axis) components, whereas the jerk is computed as the 2-norm of its longitudinal and transverse components such that $|j_{xy}|=\sqrt{{\dot{a}}_{x}^{2}+{\dot{a}}_{y}^{2}}$ . In Figure 10, notice that the inexperienced operator exhibits larger fluctuations in such signals, indicating a less smooth motion and a higher frequency of abrupt corrective actions during manual control of the mobile robot.

Figure 11 illustrates the temporal evolution of both operators during the assisted manual condition. In this case, the differences between participants are considerably reduced. Both operators rapidly achieve positioning errors e<0.05 m and exhibit smoother and more stable trajectories than those observed in the non-assisted condition.

The improvement is particularly evident for the inexperienced operator, who substantially reduces the oscillations observed previously and performs fewer corrective actions during the final approach phase. The linear and angular velocity signals evolve more progressively and with fewer abrupt changes, indicating a more stable control of the mobile robot.

Although the experienced operator still exhibits slightly smoother and more efficient trajectories, the overall differences between participants are considerably smaller than in the non-assisted condition. Similarly, the acceleration and jerk profiles show a clear reduction in dynamic fluctuations for both operators, reflecting smoother and more natural movements throughout the task.

Figure 12 presents the non-assisted teleoperation condition, where the largest differences between participants and the most unstable dynamic behavior among all evaluated conditions can be observed.

The experienced operator progressively stabilizes the mobile robot and achieves positioning errors below 0.5 m after the initial correction phase. Although small oscillations remain present, the overall motion remains relatively controlled throughout most of the task.

In contrast, the inexperienced participant exhibits pronounced oscillations in both lateral positioning error and robot heading. Furthermore, the linear and angular velocity signals show abrupt variations and frequent changes in direction, indicating a large number of overcorrections during teleoperation.

These observations suggest that this operator experiences greater difficulty simultaneously interpreting the robot dynamics, the remote interface and the spatial relationship between the manipulator and the target. As a result, the motion is considerably less stable and more erratic than that observed for the experienced operator.

This behavior is also reflected in the acceleration and jerk profiles, where the inexperienced participant exhibits larger fluctuations and abrupt dynamic changes, characteristic of a reactive and poorly stabilized control strategy.

Finally, Figure 13 presents the behavior of both operators during the assisted teleoperation condition. The incorporation of audiovisual assistance produces a substantial improvement, particularly for the inexperienced operator. The oscillations observed previously are drastically reduced and both participants achieve and maintain positioning errors e<0.05 m more rapidly and consistently.

The trajectories evolve more progressively, while the linear and angular velocity signals show a clear reduction in the abrupt corrections observed under non-assisted teleoperation.

Although the experienced operator continues to exhibit slightly smoother behavior, the dynamic differences between participants are substantially reduced compared with the previous condition. Likewise, the acceleration and jerk signals show a marked reduction in dynamic variability, particularly for the inexperienced operator, indicating smoother, more continuous and better-controlled teleoperation.

#### 4.4.3 User perception of the audiovisual assistance system

Participants reported a clearly positive perception of the audiovisual assistance system. All participants indicated that they would prefer to use some form of assistance during the positioning task. In particular, 85% expressed a preference for the complete audiovisual assistance system, whereas the remaining 15% preferred a visual-only assistance mode.

When asked which type of feedback provided a greater sense of control during task execution, participants showed a clear preference for visual information over auditory cues. This preference was evaluated using a 7-point scale, where a value of 1 indicated a complete preference for auditory feedback and a value of 7 indicated a complete preference for visual feedback. The mean score was 5.55±1.47 , suggesting that visual references were perceived as the primary source of guidance during positioning.

Nevertheless, participants also highlighted the complementary role of auditory feedback, particularly during fine positioning adjustments and in situations requiring attention shifts between the robot and the surrounding environment.

Importantly, the auditory cues were perceived as minimally intrusive. Participants reported low levels of interference with concentration (1.85±1.31) and low levels of discomfort during task execution (2.15±1.73) . These findings suggest that the auditory component can effectively complement visual feedback without increasing cognitive burden or negatively affecting the user experience.

#### 4.4.4 Perceived workload (NASA-TLX)

Subjective workload was assessed using the NASA-TLX, considering the dimensions of mental demand, physical demand, temporal demand, perceived performance, effort and frustration (Hart and Staveland, 1988). Table 2 summarizes the mean scores obtained for each dimension under the four experimental conditions.

**TABLE 2:** Mean and standard deviation of NASA-TLX workload dimensions for manual and teleoperated operation with and without audiovisual assistance.

Dimensions	Manual	Manual + Assistance	Teleoperation	Teleoperation + Assistance
Mental demand	6.8±2.31	3.55±2.35	6.2±2.71	3.65±2.58
Physical demand	5.8±2.26	5.35±1.98	2.45±2.11	1.95±1.54
Temporal demand	5.15±2.60	3.60±2.28	5.60±2.21	3.45±2.04
Perceived performance	5.6±2.11	4.5±3.47	5.1±2.13	5.4±3.27
Effort	6.55±1.73	4.20±1.54	4.65±2.46	3.35±1.98
Frustration	4.8±2.44	2.15±1.84	4.65±2.80	2.5±1.67

Audiovisual assistance produced a clear reduction in mental demand across both interaction modalities. In the manual condition, the score decreased from 6.8±2.31 to 3.55±2.35 when assistance was available. Similarly, in the teleoperated condition, mental demand decreased from 6.2±2.71 to 3.65±2.58 . The ANOVA revealed a significant effect of audiovisual assistance on this dimension (p<0.001) , whereas no significant effect of interaction mode was observed (p=0.656) .

Physical demand exhibited a different behavior from the remaining dimensions. Significantly higher scores were observed during manual operation than during teleoperation. In the manual conditions, participants reported scores of 5.8±2.26 and 5.35±1.98 , whereas the corresponding teleoperated conditions yielded substantially lower scores of 2.45±2.11 and 1.95±1.54 . Statistical analysis confirmed a significant effect of interaction mode on physical demand (p<0.001) , while the effect of audiovisual assistance was not significant (p=0.095) .

Temporal demand consistently decreased when audiovisual assistance was available. In the manual condition, the score decreased from 5.15±2.60 to 3.60±2.28 , whereas in the teleoperated condition it decreased from 5.60±2.21 to 3.45±2.04 . The ANOVA showed a significant effect of audiovisual assistance on temporal demand (p=0.003) .

Audiovisual assistance also produced a substantial reduction in perceived effort. In the manual condition, effort decreased from 6.55±1.73 to 4.20±1.54 , whereas in the teleoperated condition it decreased from 4.65±2.46 to 3.35±1.98 . Statistical analysis revealed significant effects of both interaction mode (p=0.004) and audiovisual assistance (p<0.001) .

Finally, frustration levels were markedly reduced under assisted conditions. In the manual mode, frustration decreased from 4.8±2.44 to 2.15±1.84 , whereas in the teleoperated mode it decreased from 4.65±2.80 to 2.5±1.67 . The ANOVA revealed a significant effect of audiovisual assistance (p=0.002) , while no significant effect of interaction mode was observed (p=0.768) .

Overall, these results indicate that audiovisual assistance not only improves objective task performance but also reduces the perceived cognitive burden associated with beam stopper positioning. The most pronounced effects were observed in mental demand, temporal demand, effort, and frustration, suggesting that the proposed assistance system facilitates task execution and increases user confidence during both manual and teleoperated operation.

## 5 Discussion

### 5.1 Effectiveness of the audiovisual assistance system

The primary objective of this work was to evaluate whether audiovisual guidance could improve the positioning performance of a beam stopper during both manual and teleoperated operation. The results demonstrate that the proposed assistance system significantly enhances positioning accuracy under all evaluated conditions. In particular, the incorporation of audiovisual feedback led to an approximate 80% reduction in positioning error compared with non-assisted operation, while simultaneously reducing inter-participant variability.

The NASA-TLX results further support this interpretation. Participants consistently reported lower mental demand, temporal demand, effort, and frustration when audiovisual assistance was available. These reductions indicate that the proposed feedback system not only improves objective positioning performance but also decreases the cognitive resources required to perform the task. Consequently, users can achieve higher positioning accuracy while perceiving the task as less demanding and less stressful.

An important finding is that this improvement was achieved without increasing task completion time. Although audiovisual feedback introduced an additional information channel, participants were able to use the assistance effectively without incurring a temporal penalty. This suggests that the proposed guidance strategy does not increase operational workload and instead facilitates the identification of the target location during the positioning process.

The reduction in both mean error and variability indicates that the audiovisual assistance provides robust spatial cues that improve the user’s ability to estimate the relative position between the manipulator and the target. Consequently, the system not only increases positioning accuracy but also promotes more consistent performance across users with different skill levels and operating conditions.

### 5.2 Teleoperation as a viable alternative to manual operation

The comparison between manual and teleoperated operation revealed that interaction mode has a significant influence on task execution time but not on final positioning accuracy. Participants required more time to complete the task under teleoperation, reflecting the additional cognitive demands associated with remote control, including indirect perception of the environment and delayed interpretation of robot motion.

Despite this increase in execution time, teleoperated operation achieved positioning accuracies comparable to those obtained during direct manual operation. This finding is particularly relevant because it suggests that remote positioning can be performed without sacrificing precision.

From a practical perspective, these results support the feasibility of teleoperation for beam stopper positioning tasks in radiotherapy environments. The possibility of maintaining positioning accuracy while operating remotely may contribute to improving operator safety.

Furthermore, the results indicate that audiovisual assistance remains equally effective under teleoperation, suggesting that the proposed feedback strategy can compensate for some of the perceptual limitations inherently associated with remote robot control.

### 5.3 Influence of workspace geometry on positioning performance

The analyses of workspace regions and manipulator geometry provide additional insight into the factors affecting positioning performance. In the absence of audiovisual assistance, certain target locations consistently produced larger positioning errors and greater variability between participants. These locations corresponded to configurations characterized by larger beam inclination angles and greater lateral distances between the manipulator and the target.

Such geometric configurations are likely to increase the difficulty of spatial estimation because the projected beam becomes more sensitive to small positioning errors and visual perception of the beam location becomes less intuitive. Consequently, participants were required to perform a greater number of corrective actions before converging to the target position, leading to larger errors and increased variability.

The statistical analyses confirmed this observation. Workspace region significantly affected positioning accuracy during manual operation without assistance, whereas the effect disappeared when audiovisual guidance was available. This result suggests that the observed spatial differences are primarily associated with limitations in human spatial perception rather than with the mechanical capabilities of the robotic platform itself.

One of the most notable findings of this study is that audiovisual assistance largely eliminated the influence of manipulator geometry on task performance. Even in the most demanding configurations, characterized by large inclination angles and lateral displacements, positioning accuracy remained comparable to that obtained in more favorable regions. Therefore, the proposed assistance system appears capable of compensating for the perceptual difficulties introduced by unfavorable geometric conditions.

### 5.4 Human factors and user adaptation

The influence of prior joystick experience highlights the importance of human factors in teleoperated robotic systems. Although experience did not significantly affect final positioning accuracy, it had a substantial impact on task completion time. Participants with previous teleoperation experience completed the task considerably faster than inexperienced users, particularly under teleoperated conditions.

The temporal analysis of representative operators provides additional insight into this behavior. Experienced users exhibited smoother trajectories, fewer corrective actions and lower fluctuations in velocity, acceleration and jerk signals. In contrast, inexperienced operators frequently generated oscillatory behaviors and overcorrections, particularly during teleoperation without assistance.

These observations suggest that experienced users develop more stable control strategies and more efficient interpretations of the robot response. However, the incorporation of audiovisual assistance substantially reduced these differences. Under assisted conditions, inexperienced participants exhibited smoother trajectories and fewer oscillations, approaching the performance characteristics of experienced operators.

This reduction in performance disparity is particularly relevant from a usability perspective. The results indicate that the proposed assistance system lowers the skill requirements associated with robotic positioning tasks, potentially facilitating adoption by operators with limited prior teleoperation experience.

The subjective evaluation further reinforces these findings. Participants unanimously expressed a preference for using some form of assistance during the task, with most users favoring the complete audiovisual feedback system. Although visual information was perceived as the primary source of guidance, auditory feedback was considered a useful complementary cue, particularly during fine positioning adjustments.

The NASA-TLX results are consistent with these preferences. Audiovisual assistance significantly reduced mental demand, temporal demand, effort and frustration across both interaction modalities. Importantly, these benefits were achieved without increasing physical demand and were accompanied by substantial improvements in positioning accuracy. Taken together, these findings suggest that the proposed multimodal interface not only enhances performance but also improves the overall user experience by reducing cognitive workload and facilitating more intuitive interaction with the robotic system.

Beyond the specific IOERT case study, the assistance principle investigated here is relevant to other human–robot interaction tasks in which an operator must position a device relative to an occluded or indirectly inferred target. Examples include teleoperated inspection in visually constrained environments, industrial alignment and maintenance tasks, and other medical-robotic procedures in which geometric target information can be estimated from sensors but is not directly visible to the operator. The transferable contribution is therefore not the beam-stopper hardware itself, but the conversion of continuously estimated geometric error into complementary visual and auditory cues while preserving operator authority. Future studies should determine how the cue design and threshold structure need to be adapted to the safety requirements, sensing uncertainty and workflow constraints of each application domain.

### 5.5 Limitations and future work

Several limitations should be considered when interpreting the results of this study. First, the experiments were conducted in a controlled laboratory environment that does not fully replicate the complexity of a real radiotherapy treatment room. Although the experimental setup was designed to reproduce the relevant positioning task, additional validation in clinically representative environments will be necessary before deployment in practice.

The participant cohort also limits the clinical generalizability of the human-factors results. The 20 participants were a non-clinical convenience sample drawn from the academic community, including engineering students and university staff, rather than radiation oncologists, medical physicists or other professionals who routinely work in IOERT environments. Their technical background may facilitate familiarity with robotic systems or spatial/engineering concepts, while specialized clinical personnel may differ in spatial-reasoning strategies, familiarity with accelerator geometry, baseline stress, divided-attention demands and tolerance to workload. The voluntary, uncompensated recruitment within a single academic environment may also introduce self-selection and sampling bias. Consequently, the reported NASA-TLX scores and user-adaptation effects should be interpreted as evidence for the usability of the assistance concept in this engineering-academic sample, not as direct estimates of workload in clinical staff. Future validation will therefore recruit specialized clinical personnel and explicitly assess professional experience, workflow integration, usability and ergonomics under clinically representative conditions.

A second translational issue concerns auditory feedback in the operating room. Perioperative environments already contain numerous medical-device alarms and substantial background noise, and excessive or poorly differentiated alarms can contribute to alarm fatigue and delayed responses (Ruskin and Hueske-Kraus, 2015; Konkani et al., 2012). The proposed navigation cues must therefore be designed so that they cannot mask, resemble or compete with safety-critical medical alerts. Before clinical use, the auditory channel should undergo formal acoustic and human-factors validation under representative operating-room noise, including tests of alarm audibility and identification, spectral and temporal separation from existing alerts, and workload under concurrent alarm conditions. Open-ear or spatialized delivery, conservative cue rates and context-dependent muting/adaptation may be preferable to continuously adding another alarm-like signal.

The primary group-level performance analysis in this study focuses on terminal positioning error and task completion time. The temporal analysis of representative operators additionally shows differences in oscillations, corrective actions, velocity, acceleration and jerk, but it does not provide group-level continuous-error metric. A useful extension would be to quantify the normalized integral absolute error, $IAE=\int_{0}^{T}|e\left(t\right)|\;dt$ , together with trajectory efficiency, for example the ratio between the shortest start-to-target distance and the actual traveled path length. Reporting these measures across all participants would allow the real-time stabilization benefit of audiovisual assistance to be evaluated independently of the final endpoint and will be incorporated in future experiments.

Finally, the proposed assistance system relies on relatively simple visual and auditory cues. While these cues proved highly effective, future work could investigate more advanced guidance strategies, including augmented reality interfaces, spatialized audio feedback, adaptive multimodal assistance and higher levels of shared autonomy. Such approaches may further reduce operator workload and improve positioning performance in complex clinical scenarios.

Overall, the results demonstrate that audiovisual guidance constitutes an effective and robust solution for improving beam stopper positioning performance. By reducing positioning error, mitigating the effects of unfavorable manipulator geometries and decreasing the influence of user experience, the proposed system represents a promising step toward more accessible and reliable robotic assistance in radiotherapy applications.

## 6 Conclusion

This work presented and evaluated an audiovisual assistance system designed to support the positioning of a robotic beam stopper during both manual and teleoperated operation. The proposed approach combines visual and auditory feedback to provide intuitive guidance regarding the relative position between the manipulator and the target location.

The experimental results demonstrated that the incorporation of audiovisual assistance significantly improves positioning performance. Across all evaluated conditions, the assistance system reduced the final positioning error by approximately 80% while maintaining similar task completion times. In addition, NASA-TLX assessments revealed significant reductions in mental demand, temporal demand, perceived effort and frustration, indicating that the proposed system improves accuracy while simultaneously reducing cognitive workload.

The comparison between manual and teleoperated operation showed that both interaction modalities achieve comparable positioning accuracy. Although teleoperation required longer execution times, the absence of significant differences in final positioning error suggests that remote operation constitutes a viable alternative for beam stopper positioning tasks.

The analyses of workspace regions and manipulator geometry revealed that positioning accuracy is strongly influenced by unfavorable spatial configurations when no assistance is available. However, these effects were largely mitigated through the use of audiovisual feedback. The assistance system substantially reduced the influence of beam inclination, lateral displacement and workspace location, resulting in more homogeneous performance throughout the workspace.

The study also highlighted the role of human factors in robotic positioning tasks. While prior joystick experience significantly affected task completion time, its influence on positioning accuracy was limited. Furthermore, audiovisual assistance reduced the performance gap between experienced and inexperienced operators, suggesting that the proposed system lowers the skill requirements associated with teleoperation.

Overall, the results demonstrate that audiovisual guidance provides an effective and robust solution for improving robotic beam stopper positioning. Because the present evidence was obtained from 20 non-clinical participants from the academic engineering community in a controlled laboratory environment, these conclusions concern the experimental assistance concept and should not be interpreted as clinical validation. Future work will focus on validating the proposed approach in more realistic clinical environments, including radiation oncologists, medical physicists and other relevant clinical personnel, explicitly evaluating operating-room alarm compatibility and continuous trajectory-level performance, and investigating advanced guidance strategies based on adaptive multimodal feedback, augmented reality and shared-autonomy frameworks.

## Data Availability

The raw data supporting the conclusions of this article will be made available by the authors, without undue reservation.

## References

[B1] Abbink D. A. Carlson T. Mulder M. Winter J. C. D. Aminravan F. Gibo T. L. (2018). A topology of shared control systems-finding common ground in diversity. IEEE Trans. Human-Machine Syst. 48, 509–525. 10.1109/THMS.2018.2791570

[B2] Cornejo G. Pikarsky A. Hubert A. Wygoda M. Skripai A. Popovtzer A. (2026). Neoadjuvant sbrt and intraoperative electron radiotherapy in pancreatic cancer resection. J. Cancer Res. Clin. Oncol. 152, 19. 10.1007/s00432-025-06397-2 41430468 PMC12722610

[B3] Dupont P. E. Nelson B. J. Goldfarb M. Hannaford B. Menciassi A. O’Malley M. K. (2021). A decade retrospective of medical robotics research from 2010 to 2020. Sci. Robotics 6, eabi8017. 10.1126/scirobotics.abi8017 34757801 PMC8890492

[B4] Enayati N. De Momi E. Ferrigno G. (2016). Haptics in robot-assisted surgery: challenges and benefits. IEEE Rev. Biomed. Eng. 9, 49–65. 10.1109/RBME.2016.2538080 26960228

[B5] Erdemoglu E. Ostby S. A. Senthilkumar S. Kumar A. Vora S. A. Chen L. (2025). Intraoperative radiation therapy (iort) in gynecologic cancers: a scoping review. Cancers 17, 1356. 10.3390/cancers17081356 40282536 PMC12025500

[B6] Field A. P. (2024). Discovering Statistics Using IBM SPSS Statistics. 6th edn. London, United Kingdom: SAGE Publications.

[B7] Girbes V. Armesto L. Dols J. Tornero J. (2016). Haptic feedback to assist bus drivers for pedestrian safety at low speed. IEEE Trans. Haptics 9, 345–357. 10.1109/TOH.2016.2531686 26915132

[B8] Girbes-Juan V. Schettino V. Demiris Y. Tornero J. (2021). Haptic and visual feedback assistance for dual-arm robot teleoperation in surface conditioning tasks. IEEE Trans. Haptics 14, 44–56. 10.1109/TOH.2020.3004388 32746376

[B9] Girbés-Juan V. Schettino V. Gracia L. Solanes J. E. Demiris Y. Tornero J. (2022). Combining haptics and inertial motion capture to enhance remote control of a dual-arm robot. J. Multimodal User Interfaces 16 (2), 219–238. 10.1007/S12193-021-00386-8

[B10] Goodrich M. A. Schultz A. C. (2008). Human–robot interaction: a survey. Found. Trends Human-Computer Interact. 1, 203–275. 10.1561/1100000005

[B11] Grimm A. Wollmann E. Sperk E. Weiß C. Sütterlin M. Berlit S. (2024). Intraoperative radiotherapy (iort) of early breast cancer with low-energy x-rays in breast-conserving surgery: prospective identification of pre- and intraoperative factors influencing the feasibility of iort. Strahlenther. Onkol. 200, 296–305. 10.1007/s00066-023-02149-8 37792017 PMC10965701

[B12] Hart S. G. Staveland L. E. (1988). Development of nasa-tlx (task load index): results of empirical and theoretical research. Adv. Psychol. 52, 139–183. 10.1016/S0166-4115(08)62386-9

[B13] Hensley F. W. (2017). Present state and issues in iort physics. Radiat. Oncol. 12, 37. 10.1186/s13014-016-0754-z 28193241 PMC5307769

[B14] Konkani A. Oakley B. Bauld T. J. (2012). Reducing hospital noise: a review of medical device alarm management. Biomed. Instrum. & Technol. 46, 478–487. 10.2345/0899-8205-46.6.478 23171194

[B15] Losey D. P. McDonald C. G. Battaglia E. O’Malley M. K. (2018). A review of intent detection, arbitration, and communication aspects of shared control for physical human–robot interaction. Appl. Mech. Rev. 70, 010804. 10.1115/1.4039145

[B16] Mazeas D. Namoano B. (2025). Study of visualization modalities on industrial robot teleoperation for inspection in a virtual co-existence space. Virtual Worlds 4, 17–24. 10.3390/VIRTUALWORLDS4020017

[B17] Niewels F. Knoop S. Jordan R. Michalke T. (2013). In-vehicle Sensors. 10.1002/9781118354179.auto175

[B18] Pan J. Eden J. Oetomo D. Johal W. (2024). Effects of shared control on cognitive load and trust in teleoperated trajectory tracking. IEEE Robotics Automation Lett. 9, 5863–5870. 10.1109/LRA.2024.3396111

[B19] Parallax Inc (2013). Eddie Robot Platform, (Assembled: 28990/Unassembled: 28992) v1.3.

[B20] ROBOTIS Co. Ltd. (2017). Openmanipulator-x Hardware Specification.

[B21] Roeder F. Morillo V. Saleh-Ebrahimi L. Calvo F. A. Poortmans P. Albiach C. F. (2020). Intraoperative radiation therapy (iort) for soft tissue sarcoma – estro iort task force/acrop recommendations. Radiotherapy Oncol. 150, 293–302. 10.1016/j.radonc.2020.07.019 32679306

[B22] Ruskin K. J. Hueske-Kraus D. (2015). Alarm fatigue: impacts on patient safety. Curr. Opin. Anaesthesiol. 28, 685–690. 10.1097/ACO.0000000000000260 26539788

[B23] Said S. Gozdzik M. Roche T. R. Braun J. Rössler J. Kaserer A. (2020). Validation of the raw national aeronautics and space administration task load index (nasa-tlx) questionnaire to assess perceived workload in patient monitoring tasks: pooled analysis study using mixed models. J. Med. Internet Res. 22, e19472. 10.2196/19472 32780712 PMC7506540

[B24] Sheridan T. B. (1992). Telerobotics, Automation, and Human Supervisory Control, 393.

[B25] Siciliano B. Sciavicco L. Villani L. Oriolo G. (2009). Robotics: Modelling, Planning and Control. London: Springer. 10.1007/978-1-84628-642-1

[B26] S.I.T. Sordina IORT Technologies S.p.A (2026). Liac hwl® Mobile Electron Linear Accelerator for Intraoperative Radiotherapy: User and Technical Manual.

[B27] Soriani A. Felici G. Fantini M. Paolucci M. Borla O. Evangelisti G. (2010). Radiation protection measurements around a 12 mev mobile dedicated iort accelerator. Med. Phys. 37, 995–1003. 10.1118/1.3298012 20384235

[B28] Su H. Qi W. Chen J. Yang C. Sandoval J. Laribi M. A. (2023). Recent advancements in multimodal human–robot interaction. Front. Neurorobotics 17, 1084000. 10.3389/FNBOT.2023.1084000 37250671 PMC10210148

[B29] Tecco A. D. Leonardis D. Frisoli A. Loconsole C. (2025). Grasping task in teleoperation: impact of virtual dashboard on task quality and effectiveness. Robotics 14, 92. 10.3390/ROBOTICS14070092

[B30] Wang X. Guo S. Xu Z. Zhang Z. Sun Z. Xu Y. (2024). A robotic teleoperation system enhanced by augmented reality for natural human–robot interaction. Cyborg Bionic Syst. 5, 0098. 10.34133/CBSYSTEMS.0098 39670176 PMC11636702

[B31] Winkler P. Odreitz-Stark S. Haas E. Thalhammer M. Partl R. (2020). Commissioning, dosimetric characterization and machine performance assessment of the liac hwl mobile accelerator for intraoperative radiotherapy. Z. für Med. Phys. 30, 279–288. 10.1016/j.zemedi.2020.06.004 32682654

